# Causal evidence of a line attractor encoding an affective state

**DOI:** 10.1038/s41586-024-07915-x

**Published:** 2024-08-14

**Authors:** Amit Vinograd, Aditya Nair, Joseph H. Kim, Scott W. Linderman, David J. Anderson

**Affiliations:** 1 Division of Biology and Biological Engineering, California Institute of Technology, Pasadena, CA USA; 2 Tianqiao and Chrissy Chen Institute for Neuroscience Caltech, Pasadena, CA USA; 3 Howard Hughes Medical Institute, Chevy Chase, MD USA; 4 Department of Statistics, Stanford University, Stanford, CA USA; 5 Wu Tsai Neurosciences Institute, Stanford University, Stanford, CA USA

**Keywords:** Dynamical systems, Neural circuits, Aggression, Multiphoton microscopy, Ca2+ imaging

## Abstract

Continuous attractors are an emergent property of neural population dynamics that have been hypothesized to encode continuous variables such as head direction and eye position^1–4^. In mammals, direct evidence of neural implementation of a continuous attractor has been hindered by the challenge of targeting perturbations to specific neurons within contributing ensembles^2,3^. Dynamical systems modelling has revealed that neurons in the hypothalamus exhibit approximate line-attractor dynamics in male mice during aggressive encounters^5^. We have previously hypothesized that these dynamics may encode the variable intensity and persistence of an aggressive internal state. Here we report that these neurons also showed line-attractor dynamics in head-fixed mice observing aggression^6^. This allowed us to identify and manipulate line-attractor-contributing neurons using two-photon calcium imaging and holographic optogenetic perturbations. On-manifold perturbations yielded integration of optogenetic stimulation pulses and persistent activity that drove the system along the line attractor, while transient off-manifold perturbations were followed by rapid relaxation back into the attractor. Furthermore, single-cell stimulation and imaging revealed selective functional connectivity among attractor-contributing neurons. Notably, individual differences among mice in line-attractor stability were correlated with the degree of functional connectivity among attractor-contributing neurons. Mechanistic recurrent neural network modelling indicated that dense subnetwork connectivity and slow neurotransmission^7^ best recapitulate our empirical findings. Our work bridges circuit and manifold levels^3^, providing causal evidence of continuous attractor dynamics encoding an affective internal state in the mammalian hypothalamus.

## Abstract (web summary)

Single-cell optogenetic stimulation and calcium imaging experiments provide direct evidence of line-attractor dynamics, including functional network connectivity, in the mammalian hypothalamus.

## Main

Neural circuit function has been studied from two vantage points. One focuses on understanding behaviourally specialized neuron types and their functional connectivity^8–10^, whereas the other investigates emergent properties of neural networks, such as attractors^1,3,11^. Continuous attractors of different topologies are theorized to encode a variety of continuous variables, ranging from head direction^12^, location in space^2, reward history14^ to internal states^5^. Recent data-driven methodologies have allowed for the identification of such attractor-mediated computations directly in neural data^5,13–16^. Consequently, attractor dynamics have received increasing attention as a major type of neural coding mechanism^2–4,12,13^.

Despite this progress, establishing that these attractors arise from the dynamics of the observed network remains a formidable challenge^2–4^. This calls for combining large-scale recordings with perturbations of neuronal activity in vivo. Although this has been accomplished for a point attractor that controls motor planning in cortical area anterolateral motor cortex^17,18^, spatial ensembles in visual cortex that encode visually guided behaviours^19,20^ and for a ring attractor in *Drosophila*^21,22^, there is no study reporting such perturbations for a continuous attractor in any mammalian system. While theoretical work on continuous attractors in mammals is well developed^2^, the lack of direct, neural-perturbation-based experimental evidence of such attractor dynamics has hindered progress towards a mechanistic circuit-level understanding of such emergent manifold-level network features^3^.

Oestrogen receptor type 1 (*Esr1*)-expressing neurons in the ventrolateral subdivision of the ventromedial hypothalamus (VMHvl^*Esr1*^) comprise a key node in the social behaviour network and have been causally implicated in aggression^23,24^. Calcium imaging of these neurons in freely behaving animals has revealed mixed selectivity and variable dynamics, with time-locked attack signals sparsely represented at the single-neuron level^25,26^. Application of dynamical system modelling^27^ has revealed an approximate line attractor in the VMHvl that correlates with the intensity of agonistic behaviour, suggesting a population-level encoding of a continuously varying aggressive internal state^5^. This raises the question of whether the observation of a line attractor in a statistical dynamical systems model fit to VMHvl^*Esr1*^ neuronal activity reflects inherited dynamics or can be instantiated locally.

This question can be addressed, in principle, using all-optical methods to observe and perturb line-attractor-relevant neural activity^3,28–30^. A challenge in applying these methods during aggression is that current technology requires head-fixed preparations, and head-fixed mice cannot fight. To overcome this challenge, we took advantage of a recent observation that VMHvl-progesterone receptor neurons (which encompass the *Esr1*^*+*^ subset)^31–33^ mirror observed interindividual aggression^6^, to instantiate the line attractor in head-fixed mice. Using this preparation, we performed model-guided, closed-loop on- and off-manifold perturbations^34^ of VMHvl^*Esr1*^ activity. These experiments demonstrate that the VMHvl line attractor indeed reflects causal neural dynamics in this nucleus. They also identified selective functional connectivity within attractor-weighted ensembles, suggesting a local circuit implementation of attractor dynamics. Modelling suggests that this implementation may incorporate slow neurotransmission. Collectively, our findings elucidate a circuit-level foundation for a continuous attractor in the mammalian brain.

## Line attractor for observing aggression

**Extended Data Fig. 1:**
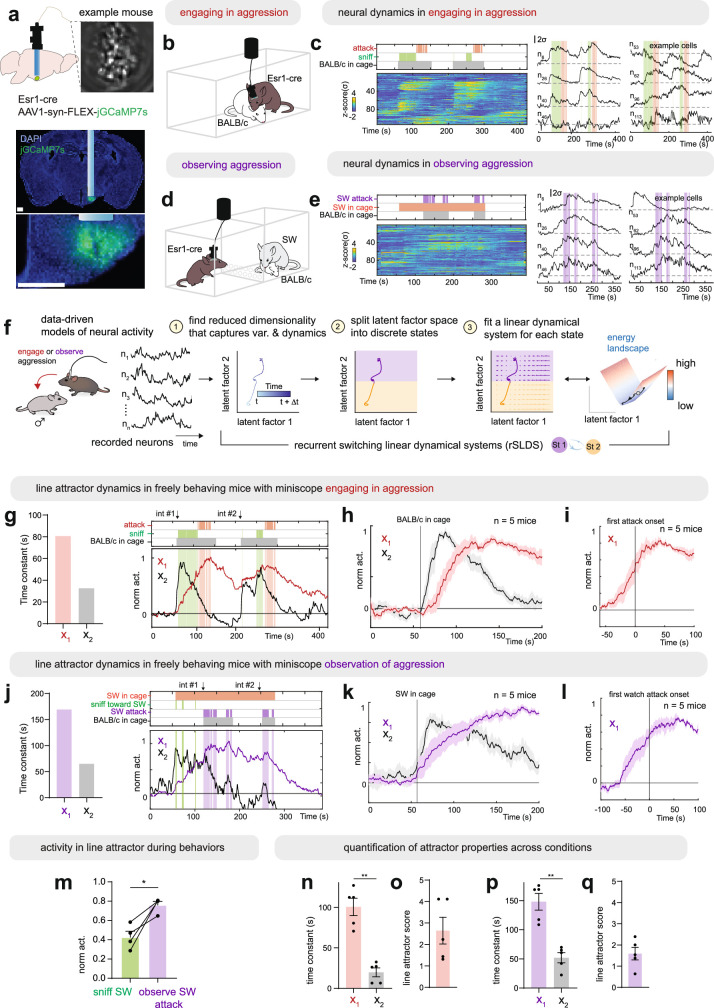
Shared line attractor dynamics in freely behaving mice engaging in or observing aggression. a. Implantation of miniscope, field of view (top) and fluorescence image showing histology (bottom) with jGCaMP7s expression in VMHvl. N = 5 mice. b. Experimental paradigm to record VMHvl^*Esr1*^ activity in mice engaging in aggression. c. Left: neural & behavioural raster of example mouse 1 when engaging in aggression. Right: example neurons. d. Experimental paradigm to record VMHvl^*Esr1*^ activity in same mice in Extended Data Fig. 1c during observation of aggression. e. Left: neural & behavioural raster of example mouse 1 during observation of aggression. Right: example neurons. f. Overview of rSLDS analysis. g. Left: rSLDS time constants in example mouse 1. Right: Normalized neural activity projected onto two dimensions (*x*_1_ and *x*_2_) of dynamical system. h. Behaviour triggered average of normalized *x*_1_ and *x*_2_ dimensions, aligned to introduction of male intruder (n = 5 mice, average trace in dark red and black ± sem in shaded area). i. Behaviour triggered average of *x*_1_ dimensions, aligned to first attack onset (n = 5 mice, average trace in dark red ± sem in shaded red area). j. Left: rSLDS time constants in example mouse 1 during observation of aggression. Right: Neural activity projected onto two dimensions (*x*_1_ and x_2_) of dynamical system. k. Behaviour triggered average of normalized *x*_1_ and *x*_2_ dimensions from observation of aggression, aligned to introduction of BALB/c into resident’s cage (n = 5 mice, average trace in dark purple and black ± sem in shaded area). l. Behaviour triggered average of *x*_1_ dimensions from observation of aggression, aligned to first bout of observing attack (n = 5 mice, average trace in dark purple ± sem in shaded purple area). m. Average activity in the *x*_1_ dimension during sniffing of the SW mouse, vs observing the SW mouse a BALB\c intruder (n = 4 mice, *p = 0.0286, Two-tailed Mann Whitney *U*-test, error bars - sem). n. rSLDS time constants across mice engaging in aggression (n = 5 mice, *p = 0.0079, Two-tailed Mann Whitney *U*-test, error bars - sem). o. Line attractor score across mice engaging in aggression (n = 5 mice, error bars - sem). p. rSLDS time constants across mice during observation of aggression (n = 5 mice, *p = 0.0079,Two-tailed Mann Whitney *U*-test, error bars - sem). q. Line attractor score across mice during observation of aggression (n = 5 mice, error bars - sem).

**Extended Data Fig. 2:**
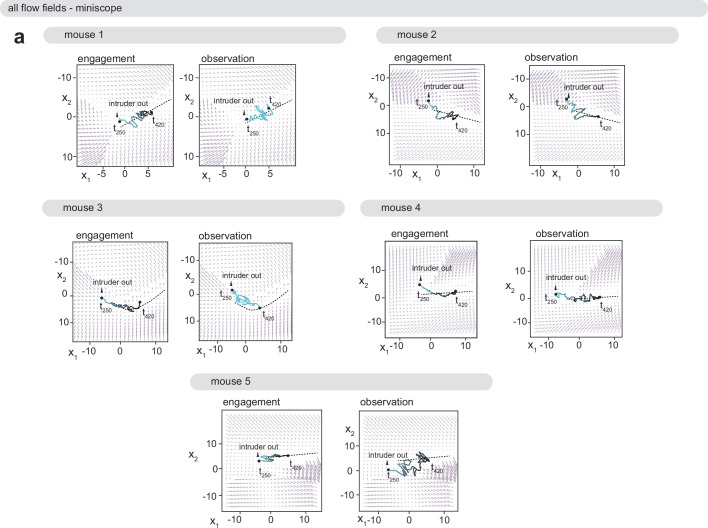
Flow fields from miniscope experiments during engagement and observation of aggression. Flow fields from all mice showing neural trajectories aligned to removal of the intruder or demonstrator mouse in either observation or engagement of aggression. Dashed lines highlight region of slow points (line attractor).

**Extended Data Fig. 3:**
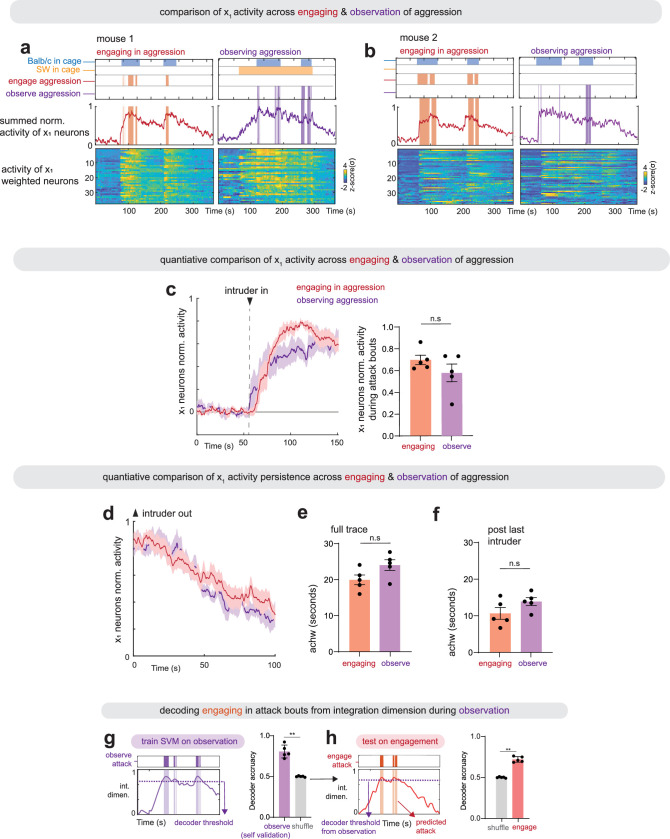
Comparing neuronal activity of *x*_*1*_ neurons during engaging vs. observing aggression. a. Normalized neuronal activity of all *x*_*1*_ neurons from example mouse 1 when engaging in aggression (left) and observing aggression (right). Bottom: Raster plots of the activity of all neurons from *x*_*1*_ dimension in mouse 1. b. Same as in panel a but for example mouse 2. c. comparing the activity of *x*_*1*_ neurons between observing and engaging in aggression. Left: Average activity across mice (n = 5 mice, shaded area is sem). Right: comparison of the activity during observing attack bouts and engaging in attack (n = 5 mice, p = 0.42, Two-tailed Mann-Whitney *U*-test, error bars - sem). d. Activity of *x*_*1*_ neurons aligned to removal of last intruder during observation and engaging in aggression (n = 5 mice, shaded area is sem). e. Quantification of autocorrelation half-width for *x*_*1*_ neurons in both conditions during the full interaction (mean achw during observation: 25 ± 0.8s, mean achw during engagement: 20 ± 1.7s, n=5 mice, p = 0.125, Two-tailed Mann-Whitney *U*-test, error bars - sem). f. Quantification of achw for *x*_*1*_ neurons in both conditions aligned to removal of last intruder (mean achw during observation: 14 ± 1s, mean achw during engagement: 11 ± 1.6s, n = 5 mice, p = 0.187, Two-tailed Mann-Whitney *U*-test, error bars - sem). g. Decoding bouts of attack during engaging in aggression from integration dimension activity during observation of attack. Left: Decoder strategy. A SVM decoder was trained on data from integration dimension activity to separate bouts of observing attack from non attack bouts. Right: Quantification of the decoder accuracy performance (n = 5 mice, p = 0.0079, Two-tailed Mann-Whitney *U*-test, error bars - sem). h. Left: Strategy for testing the decoder. The SVM decoder that was trained on observation of attack is tested with data from engaging in attack. Right: Quantification of the performance of the decoder on engaging vs shuffled data (n = 5 mice, p = 0.0079, Two-tailed Mann-Whitney *U*-test, error bars - sem).

**Extended Data Fig. 4:**
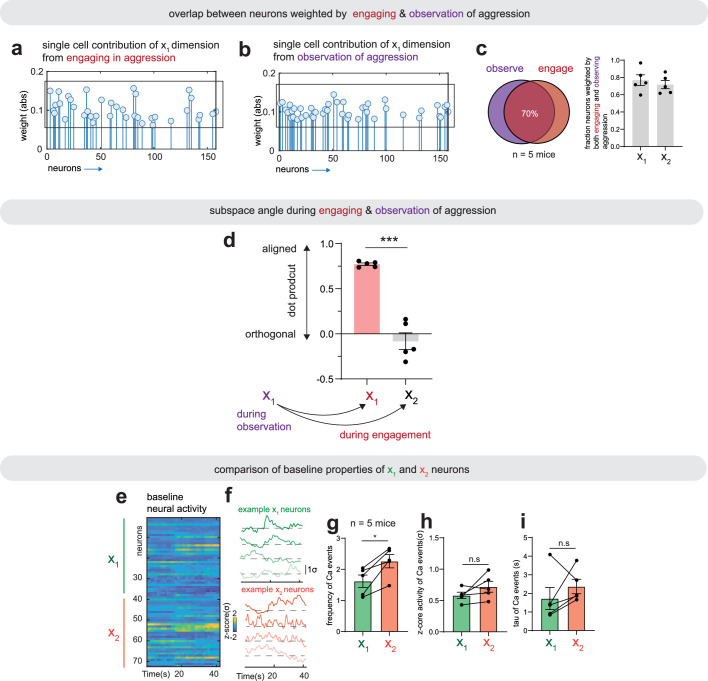
Single cell comparison of integration neurons across conditions. a. Single cell contribution of *x*_*1*_ dimension (rSLDS weights) from engagement of aggression in example mouse. b. Single cell contribution of *x*_*1*_ dimension (rSLDS weights) from observation of aggression in example mouse. c. Overlap in neurons contributing to line attractor *x*_*1*_ & *x*_*2*_ dimension from rSLDS performing independently in engaging versus observing aggression. Left: Example mouse, Right: Average across 5 mice, error bars - sem. d. Dot product of *x*_*1*_ neural weight vectors during observation vs. engagement in aggression. rSLDS weights of the *x*_*1*_ dimension during observation were compared to model weights of the *x*_*1*_ and *x*_*2*_ dimensions during engagement using a dot product of the two weight vectors. (n = 5 mice, ***p = 0.0079, Two-tailed Mann-Whitney *U*-test, error bars - sem). e. Example raster of baseline activity from one mouse freely behaving while solitary in its home cage. f. Example single-cell traces from raster in Ex. Data Fig.e. Top - *x*_*1*_ neurons, bottom - *x*_*2*_ neurons. g. Comparison of frequency of Ca^+2^ transients (above 1.5*σ* in z-score activity) during baseline recordings across mice (mean frequency *x*_*1*_: 1.6 ± 0.2 events, mean frequency *x*_*2*_: 2.3 ± 0.2 events, n = 5 mice, *p = 0.012, Two-tailed Mann-Whitney *U*-test, error bars - sem). h. Comparison of the mean amplitude of Ca^+2^ transients in *x*_*1*_ vs. *x*_*2*_ neurons during baseline recordings, averaged across mice (mean amplitude *x*_*1*_: 0.58 ± 0.04 z-score, mean amplitude *x*_*2*_: 0.71 ± 0.08 z-score, n = 5 mice, p = 0.188, Two-tailed Mann-Whitney *U*-test, error bars - sem). i. Comparison of the decay time of Ca^+2^ events during baseline recordings across mice (mean tau *x*_*1*_: 1.7 ± 0.6s, mean tau *x*_*2*_: 2.3 ± 0.4s, n = 5 mice, p = 0.34, Two-tailed Mann-Whitney *U*-test, error bars - sem).

Recent studies have demonstrated that the VMHvl contains neurons that are active during passive observation of, as well as active participation in, aggression and that reactivating the former can evoke aggressive behaviour^6^. However, those findings were based on a relatively small sample of VMHvl neurons, which might comprise a specific subset distinct from those contributing to the line attractor (the latter represent around 20–25% of *Esr1*^*+*^ neurons^5^). To assess whether these mirror-like responses can be observed in *Esr1*^*+*^ neurons that contribute to line-attractor dynamics, we performed microendoscopic imaging^35^ of VMHvl^*Esr1*^ neurons expressing jGCaMP7s in the same freely behaving animals during engagement in followed by observation of aggression (Extended Data Fig. 1a–e). Analysis using recurrent switching linear dynamical systems (rSLDS)^27^ to fit a statistical model to each dataset (Extended Data Fig. 1f) revealed an approximate line attractor under both conditions, exhibiting ramping and persistent activity aligned and maintained across both performed and observed attack sessions (Extended Data Figs. 1g–q, 2 and 3a–f). Activity during observation of aggression in the integration dimension (*x*_1_), which contributes to the line attractor, could be reliably used to decode from held-out data instances of both observation of and engagement in attack, suggesting that this dimension encodes a similar internal state variable under both conditions (Extended Data Fig. 3g,h). Moreover, the integration dimension was weighted by a consistent and aligned set of neurons under both conditions, suggesting that a highly overlapping set of neurons (70%) contributes to line-attractor dynamics during observing or engaging in attack (Extended Data Fig. 4a–d).

The dynamical systems analysis also revealed a dimension orthogonal to the integration dimension (*x*_2_) that displayed faster dynamics time locked to the entry of the intruder(s) in both conditions (Extended Data Fig. 1g–l). To examine whether the neurons contributing to the two dimensions (*x*_1_ and *x*_2_ neurons) can be separated on the basis of physiological properties, we examined their baseline activity when solitary animals were exploring their home cage before any interaction. We did not detect a difference in amplitude or decay constant (tau) between *x*_1_ and *x*_2_ neurons (Extended Data Fig. 4e–i). However, we did see a slightly but significantly higher frequency of spontaneous calcium transients in *x*_2_ neurons (Extended Data Fig. 4f,g), suggesting that *x*_2_ neurons are more spontaneously active than *x*_1_ neurons when no interaction is taking place.

While these observed attractor dynamics could be generated in the VMHvl, they might also arise from unmeasured ramping sensory input or dynamics inherited from an input brain region^36^. Although behavioural perturbations in previous studies have hinted at the intrinsic nature of VMHvl line-attractor dynamics^5^, a rigorous test requires direct neuronal perturbations^34,37^ targeted to cells that contribute to the attractor. Direct on-manifold perturbation of a continuous attractor has previously been performed only in the *Drosophila* head direction system^12,21^. In mammals, although a point attractor has been perturbed off-manifold using optogenetic manipulation^17,18,28^, direct single-cell perturbations of neurons contributing to a continuous attractor in vivo have not been reported.

**Fig. 1:**
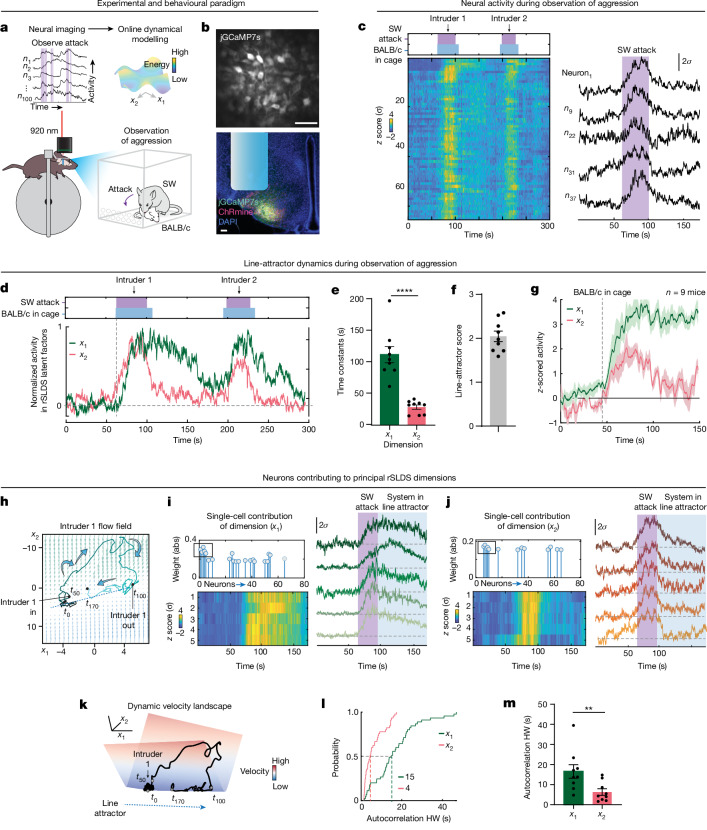
Attractor dynamics in head-fixed mice observing aggression. **a**, The experimental paradigm for 2P imaging in head-fixed mice observing aggression. **b**, Representative FOV through a GRIN lens in the 2P set-up (top). Bottom, fluorescence image of a coronal slice showing expression of jGCaMP7s and ChRmine. Scale bars, 100 µm. **c**, Neural and behavioural raster from an example mouse observing aggression in the 2P set-up (left). The arrows indicate insertion of submissive BALB/c intruders into the observation chamber for interaction with an aggressive Swiss Webster (SW) mouse. Right, example neurons from the raster to the left. **d**, Neural activity projected onto rSLDS dimensions obtained from models fit to 2P imaging data in one example mouse. **e**, rSLDS time constants across mice. *n* = 9 mice. Statistical analysis was performed using two-tailed Mann–Whitney *U*-tests. Data are mean ± s.e.m. **f**, The line-attractor score (Methods) across mice. *n* = 9 mice. Data are mean ± s.e.m. **g**, Behaviour-triggered average of *x*_1_ and *x*_2_ dimensions, aligned to the introduction of BALB/c mice into the resident’s cage. *n* = 9 mice. Data are the average activity (dark line) ± s.e.m. (shading). **h**, Flow fields from rSLDS model fit to 2P imaging data during observation of aggression from one example mouse. The larger blue arrows next to the neural trajectory indicate the direction flow of time. The smaller arrows represent the vector field from the rSLDS model. **i**, Identification of neurons contributing to *x*_1_ dimension from rSLDS model (top). The neuron’s weight is shown as an absolute (abs) value. Bottom, activity heat map of five neurons contributing most strongly to the *x*_1_ dimension. Right, neural traces of the same neurons and an indication of when the system enters the line attractor. **j**, As in **i** but for the *x*_2_ dimension. **k**, Dynamic velocity landscape from 2P imaging data during observation of aggression from one example mouse. Blue, stable area in the landscape; red, unstable area in the landscape. The black line shows the trajectory of neuronal activity. **l**, The cumulative distributions of the autocorrelation half width (ACHW) of neurons contributing to the *x*_1_ (green) and *x*_2_ (red) dimensions. *n* = 9 mice, 45 neurons each for the *x*_1_ and *x*_2_ distributions. **m**, The mean autocorrelation half width (HW) across mice for neurons contributing to the *x*_1_ and *x*_2_ dimensions. *n* = 9 mice. Statistical analysis was performed using a two-tailed Mann–Whitney *U*-test; **P = 0.0078. Data are mean ± s.e.m. ****P *<* 0.0001, **P < 0.01.

**Extended Data Fig. 5:**
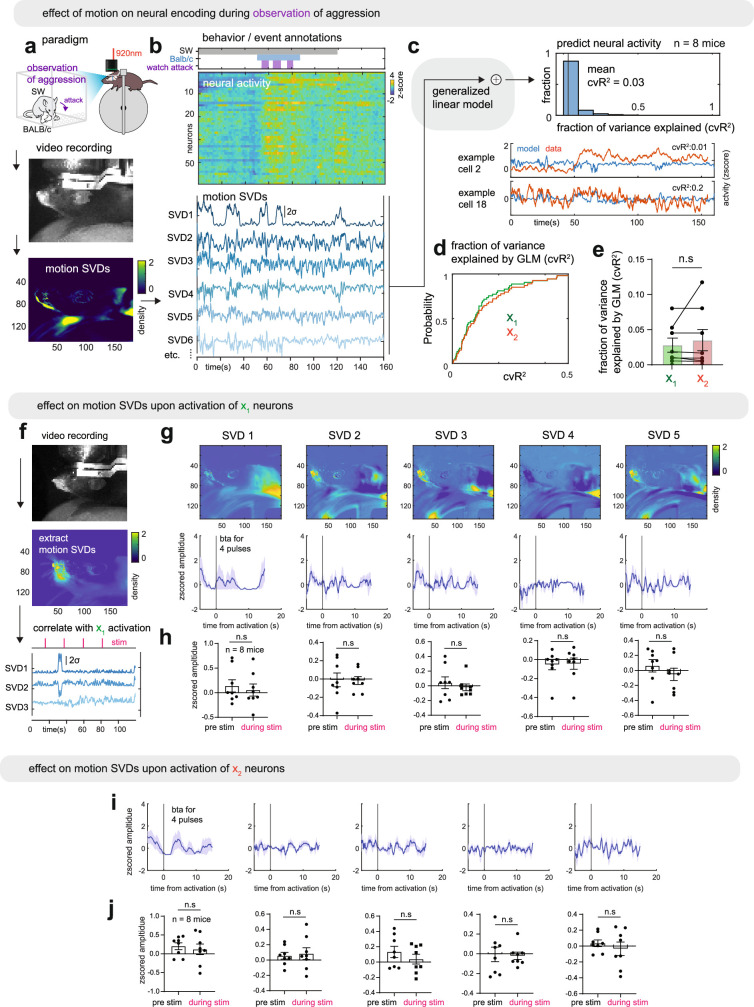
Readouts of behaviour and motion in head-fixed mice. a. Top: Experimental paradigm for 2-photon imaging in head-fixed mice observing aggression. A 920nm 2-photon laser was used to monitor activity of *Esr1*^+^ neurons in VMHvl. Middle: One frame from a video recorded during observation of aggression. Bottom: An example of one motion SVD. b. Top: Neural activity raster during observation of aggression. Bottom: examples of SVD outputs over time during observation of aggression. c. Top: Predicted neuronal activity of single neurons and their variance explained by a generalized linear model (GLM) from SVDs readout over time. Bottom: Two example cells with different levels of variance explained. d. Estimated cumulative distribution of variance explained by the GLM of either *x*_*1*_ or *x*_*2*_ neurons across all mice. e. Statistical comparison of variance explained by GLM of *x*_*1*_ activity or *x*_*2*_ activity neurons per mouse (n = 7 mice, p = 0.8125, Two-tailed Mann-Whitney *U*-test, error bars - sem). f. Top: One frame from a video recorded during group photo-activation of *x*_*1*_ neurons. Middle: An example of one motion SVD. Bottom: Time-evolving activity of top 3 SVDs aligned to *x*_*1*_ activation (vertical red bars = photoactivation pulses). g. Projection of top 5 motion SVDs and stimulus triggered average of each SVD aligned to the start of *x*_*1*_ activation. h. Average response in top 5 SVDs during pre-stimulus and stimulus periods (n = 8 mice, p >0.05, Two-tailed Mann-Whitney *U*-test, error bars - sem). i. Same as g, but for activation of *x*_*2*_ neurons. j. Same as h, but for activation of *x*_*2*_ neurons.

To do this, we used two-photon (2P) imaging in head-fixed mice of VMHvl^*Esr1*^ neurons expressing jGCaMP7s^38^ after observation of aggression and removal of the demonstrator mice (Fig. 1a–c). As described above, during observation of aggression by the head-fixed mice, rSLDS analysis identified an integration dimension with slow dynamics (*x*_1_) aligned to an approximate line attractor, and an orthogonal dimension with faster dynamics (*x*_2_) (Fig. 1d–h,k). We used the mapping between neural activity and the underlying state space to directly identify neurons contributing to each dimension (Fig. 1i,j). Neurons contributing to the integration dimension displayed more persistence than those aligned with the faster dimension (Fig. 1g,l,m). Importantly, only a small fraction of the neural activity could be explained by movements of the observer mouse (Extended Data Fig. 5a–e). Thus, a line attractor can be recapitulated in head-fixed mice observing aggression, opening the way to 2P-based perturbation experiments.

## Holographic activation shows integration

**Fig. 2:**
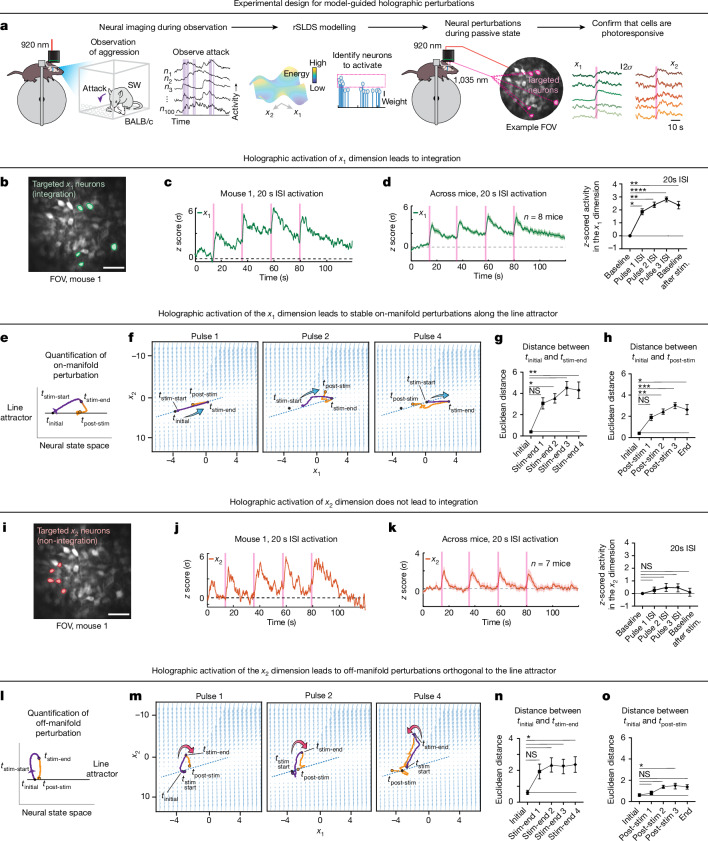
Holographic perturbations reveal integration dynamics in the VMHvl. **a**, The experimental paradigm for 2P perturbation in head-fixed mice. **b**, FOV of five *x*_1_ neurons selected for 2P activation in example mouse 1. Scale bar, 100 µm. **c**, Neural activity projected onto the *x*_1_ dimension after holographic activation of five *x*_1_ neurons in example mouse 1. The pink vertical lines show the time of activation. **d**, The average activity projected onto the *x*_1_ dimension from activation of 5 *x*_1_ neurons (left). Data are average (dark green) ± s.e.m. (shaded green area). *n* = 8 mice. Right, the average *z*-scored activity of the projected *x*_1_ dimension during the baseline or ISIs. *n* = 8 mice. Statistical analysis was performed using Kruskal–Wallis tests with Dunn’s correction; *P = 0.0363, **P = 0.0013 (bottom), **P = 0.0067 (top). Data are mean ± s.e.m. **e**, Schematic of quantifying perturbation along line attractor in neural state space. **f**, Flow fields from example mouse 1, showing perturbations along the line attractor after activation of 5 *x*_1_ neurons. The larger blue arrows next to the neural trajectory indicate the direction flow of time. The smaller arrows indicate the vector field from the rSLDS model. **g**, The Euclidian distance between time points *t*_initial_ and *t*_stim-end_ across mice. *n* = 8 mice. Statistical analysis was performed using Kruskal–Wallis tests with Dunn’s correction; not significant (NS), *P* = 0.061; *P = 0.029, **P = 0.0018 (bottom), **P = 0.0059 (top). Data are mean ± s.e.m. **h**, As in **g** but for timepoints *t*_initial_ and *t*_post-stim_. *n* = 8 mice. Statistical analysis was performed using Kruskal–Wallis tests with Dunn’s correction; NS, *P* = 0.1965; **P = 0.0082, ***P = 0.0004, *P = 0.016. Data are mean ± s.e.m. **i**, FOV of five *x*_2_ neurons selected for activation in example mouse 1. Scale bar, 100 µm. **j**, Neural activity projected onto the *x*_2_ dimension after holographic activation of *x*_2_ neurons in example mouse 1. **k**, The average activity projected onto the *x*_2_ dimension from activation of *x*_2_ neurons (left). Data are average (dark red) ± s.e.m. (shaded red area). *n* = 7 mice. Right, the average z-scored activity of the projected *x*_2_ dimension during the baseline or ISIs. *n* = 7 mice. Statistical analysis was performed using Kruskal–Wallis tests with Dunn’s correction; *P* > 0.99. Data are mean ± s.e.m. **l**, As in **e** but for *x*_2_ activation. **m**, Flow fields from example mouse 1, showing *x*_2_ activation. The red arrows indicate the direction of the flow of time. **n**, As in **g** but for *x*_2_ activation. *n* = 7 mice. Statistical analysis was performed using Kruskal–Wallis tests with Dunn’s correction; NS, *P* = 0.1554; *P = 0.042 (bottom), *P = 0.029 (middle), *P = 0.029 (top). Data are mean ± s.e.m. **o**, As in **h** but for *x*_2_ activation. *n* = 7 mice. Statistical analysis was performed using Kruskal–Wallis tests with Dunn’s correction; NS, *P* > 0.05 (bottom), *P* = 0.508 (middle), *P* = 0.0508 (top); *P = 0.0383. Data are mean ± s.e.m. NS, *P* > 0.05; *P *<* 0.05, ***P < 0.001, ****P < 0.0001.

**Extended Data Fig. 6:**
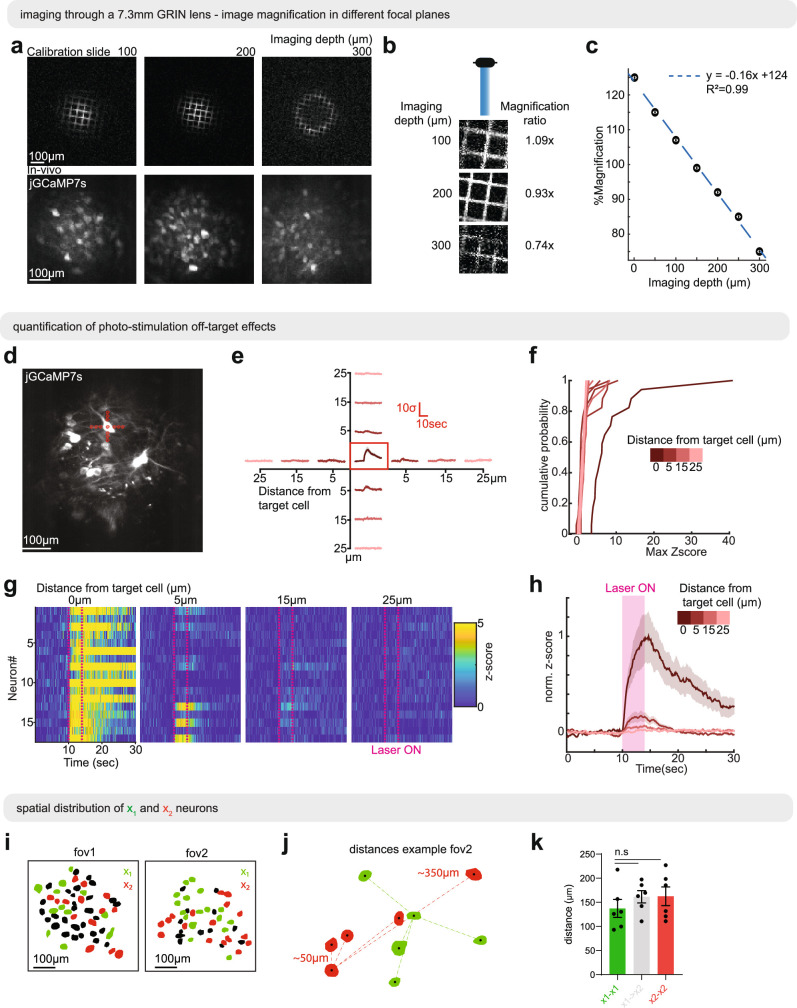
Controls for off-target effects of 2P photoactivation. a. GRIN lens changes the spatial resolution based on the axial depth. Top: imaging a calibration slide with 40 μm fluorescent squares at different axial distances below the GRIN lens. Bottom: imaging in-vivo jGCaMP7s expressing *Esr1*^+^ neurons in the VMHvl at different axial distances below the GRIN lens. b. Magnification ratio at different imaging depths calculated from the fluorescent calibration slide. c. Quantification of the relationship between imaging depth and magnification error. Linear regression is used to estimate the degree of aberration caused by the GRIN lens. d. Example field of view illustrating the experimental procedure for mapping the spatial resolution of 2P targeted photo-stimulation through the GRIN lens. Reference neurons were targeted first centred on their somata, and then again stepwise at different distances from the soma centre along each of the four cardinal directions, using 10 µm diameter stimulation spirals. N = 17 cells. e. Average response of all tested neurons to stimulation at each location from the soma. Shaded area represents standard error of the mean. The red-boxed trace indicates the response observed when the stimulation is centred on the reference cell (0 µm). f. Estimated cumulative distribution of the reference cell responses at different distances from soma. Lighter shades of red represent responses at distances progressively further from the soma. n = 17 neurons. g. Raster of neural activity of all 17 reference neurons tested using the procedure in Ex. Data Fig. e. Note that at 15 µm the average response in the reference cells is close to zero. h. Normalized average activity of all neurons at different distances from soma. Each row is a different experiment on a different reference cell. i. Representative examples of field of views from two mice. Green - all *x*_*1*_ neurons, Red – all *x*_*2*_ neurons, black - non *x*_*1*_ or *x*_*2*_ neurons. Fov - field of view. j. Example illustrating how distances are calculated for estimating the spatial clustering of *x*_*1*_ and *x*_*2*_ neurons. k. Quantification of average distance within *x*_*1*_ and *x*_*2*_ neurons and between *x*_*1*_ and *x*_*2*_ neurons, across mice (n = 8 mice, p > 0.05: Kruskal-Wallis test with Dunn’s correction for multiple comparison, error bars - sem).

**Extended Data Fig. 7:**
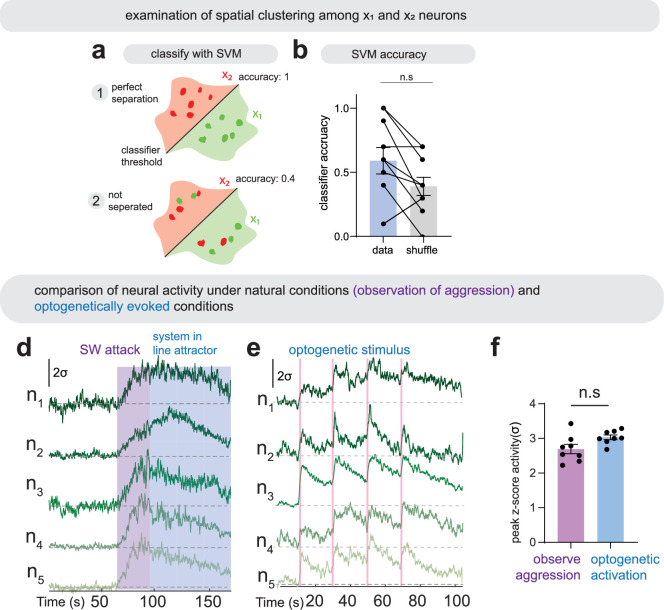
Spatial clustering of neurons and activity comparison. a. Support vector machine decoder trained to separate cell positions of *x*_*1*_ versus *x*_*2*_ neurons. Scenario 1 shows a cartoon where cells are perfectly separated by the SVM decoder and scenario 2 shows a cartoon where cells are inseparable based on their spatial location and shows low classifier accuracy. b. Accuracy of SVM decoder trained on data versus shuffled control (n=10 mice, p=0.156: Two-tailed Mann-Whitney *U*-test, error bars - sem). c. Classification width of SVM decoder trained on data versus shuffled control (n=10 mice, p=0.578: Two-tailed Mann-Whitney *U*-test). d. Neural activity of five *x*_*1*_ neurons selected for grouped optogenetic targeting during observation of aggression. e. Neural activity of the same five *x*_*1*_ neurons in panel d during grouped optogenetic activation. f. Comparison of peak z-score of *x*_*1*_ neurons selected for grouped optogenetic activation during observation of aggression and during optogenetic activation (n = 8 mice, p > 0.05: Two-tailed Mann-Whitney *U*-test, error bars - sem).

Next, to determine whether VMHvl^*Esr1*^ line-attractor dynamics are intrinsic to this hypothalamic nucleus, after removing the demonstrator mice, we performed holographic re-activation of a subset of neurons contributing to the integration dimension (*x*_1_) using soma-tagged ChRmine^39^, which was co-expressed with jGCaMP7s (Fig. 1b (bottom)). These neurons were identified in real-time using rSLDS fitting of data recorded during observation of aggression (in a manual closed loop), followed by 2P single-cell-targeted optogenetic reactivation of those neurons (Fig. 2a). In each field of view (FOV), we concurrently targeted five neurons, chosen on the basis of the criteria that they (1) contributed most strongly to a given dimension (*x*_1_ or *x*_2_); and (2) could be reliably reactivated by photostimulation (Fig. 2a). Repeated pulses of optogenetic stimulation (2 s, 20 Hz, 5 mW) were delivered with a 20 s interstimulus interval (ISI) (Fig. 2b–d). Under these conditions, we observed minimal off-target effects (Extended Data Fig. 6a–h) and did not observe spatial clustering of *x*_1_ or *x*_2_ neurons (Methods and Extended Data Figs. 6i–k and 7a,b).

**Extended Data Fig. 8:**
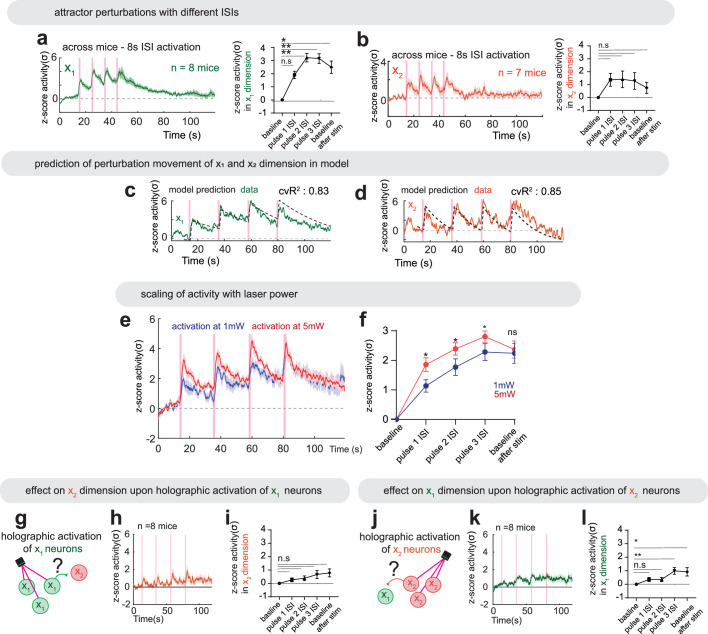
Characterization of line attractor properties. a. Average activity projected onto *x*_*1*_ dimension from activation of *x*_*1*_ neurons across mice using 8s inter stimulus interval (n = 7 mice). Shaded area – sem. Right: Quantification of average z-scored activity of projected *x*_*1*_ dimension during baseline or inter stimulus intervals (n = 7 mice, n.s p = 0.3, **p = 0.0012, **p = 0.0012, *p = 0.0192 Kruskal-Wallis test with Dunn’s correction for multiple comparison, error bars - sem). b. Average activity projected onto *x*_*2*_ dimension from activation of *x*_*2*_ neurons across mice using 8s inter stimulus interval (n = 7 mice). Shaded area – sem. Right: Quantification of average z-scored activity of projected *x*_*2*_ dimension during baseline or inter stimulus intervals (n.s p > 0.05, n = 7 mice, Kruskal-Wallis test with Dunn’s correction for multiple comparison, error bars - sem). c. Data and model prediction of applying stimulation paradigm in Fig. 2c to rSLDS model trained on observing aggression. d. Data and model prediction of applying stimulation paradigm in Fig. 2j to rSLDS model trained on observing aggression. e. *x*_*1*_ integration dimension activity with 1mW per neurons (blue) and 5mW per neuron (red). Shaded area – sem. n = 8 mice. f. Quantification of average z-scored activity of projected *x*_*1*_ dimension neurons in 1mW and 5mW per neuron during baseline or various inter stimulus intervals (n = 8 mice, *p = 0.0295, *p = 0.0186, *p = 0.045, n.s p = 0.7, Two-tailed Mann-Whitney *U*-test, error bars - sem). g. Paradigm for examining activity in *x*_*2*_ dimension upon grouped holographic activation of *x*_*1*_ neurons. h. Average z-score activity of neural activity projected onto *x*_*2*_ dimension across mice (n = 8 mice). Shaded area – sem. i. Quantification of activity in non-targeted *x*_*2*_ dimension upon grouped holographic activation of *x*_*1*_ neurons (n.s, n = 8 mice, Kruskal-Wallis test with Dunn’s correction for multiple comparison, error bars - sem). j. Paradigm for examining activity in *x*_*1*_ dimension upon grouped holographic activation of *x*_*2*_ neurons. k. Average z-score activity of neural activity projected onto *x*_*1*_ dimension across mice (n = 8 mice, Shaded area – sem). l. Quantification of activity in non-targeted *x*_*1*_ dimension upon grouped holographic activation of *x*_*2*_ neurons (n.s p = 0.276, n.s p = 0.276, **p = 0.0072, *p = 0.03, n = 8 mice, Kruskal-Wallis test with Dunn’s correction for multiple comparison, error bars - sem).

In this paradigm, optogenetically induced activity along the *x*_1_ (but not the *x*_2_) dimension is predicted to exhibit integration across successive photostimulation pulses, based on the time constants of these dimensions extracted from the fit rSLDS model (Fig. 1e). Consistent with this expectation, optogenetic reactivation of cohorts of five individual *x*_1_ neurons yielded robust integration of activity in the entire *x*_1_ dimension-weighted population, as evidenced by progressively increasing peak activity during the 20 s ISI after each consecutive pulse (Fig. 2c,d; *n* = 8 mice). Activity decayed slowly after each peak but did not return to pre-stimulus baseline. Activated *x*_1_ neurons exhibited activity levels comparable to their response during observation of aggression (Extended Data Fig. 7d–f). Similar results were obtained using an 8 s ISI (Extended Data Fig. 8a). This activity also scaled with different laser powers (Extended Data Fig. 8e,f). Providing the same (digital optogenetic) input to the fit rSLDS model also resulted in integration by the model along the *x*_1_ dimension, similar to that observed in the data (Extended Data Fig. 8c). Importantly, *x*_1_ stimulation did not evoke appreciable activity in *x*_2_ dimension neurons (Extended Data Fig. 8g–i).

**Extended Data Fig. 9:**
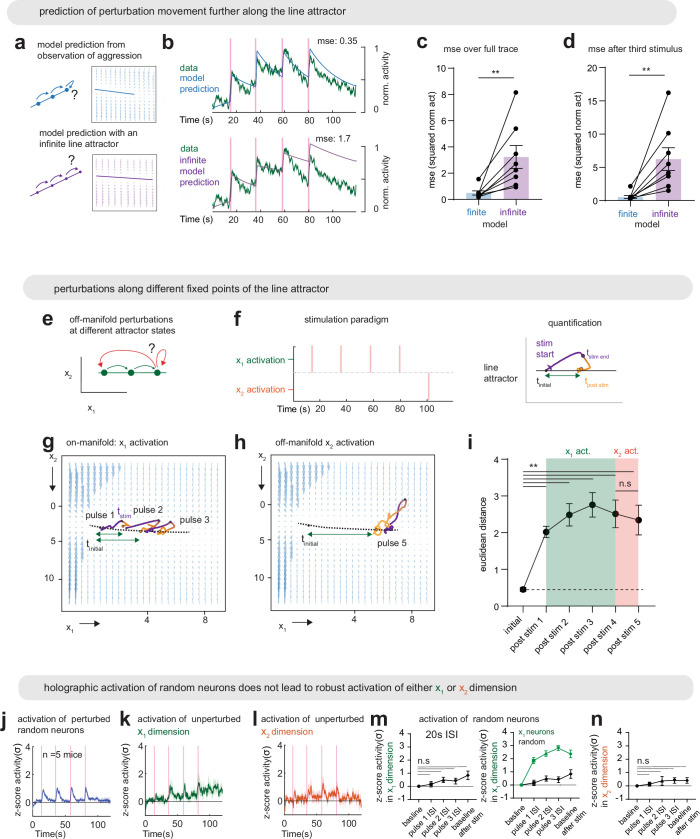
Examination of finite nature and stability of line attractor. a. Top: model prediction, assuming there is a finite length of the attractor, after the system reaches a certain point along the attractor, further pulses of activity will not cause a further ramp. Bottom: If the line attractor is infinite, then each activation should push the system further along the attractor. b. Example from one mouse comparing the prediction of finite (top) and infinite (bottom) model of the line attractor. Pink lines represent time of photoactivation. Mse - mean square error between model and the data. c. Comparison of the mse of the whole trace between the data and either the finite or infinite models (n = 8 mice, **p<0.001, Two-tailed Mann-Whitney *U*-test, error bars - sem). d. Same as Extended Data Fig. 9c but comparing only after the third pulse. Note that the scale of the y axis in Extended Data Fig. 9d is twice as big as in Extended Data Fig. 9c (n = 8 mice, **p<0.001, Two-tailed Mann-Whitney *U*-test, error bars - sem). e. Testing off-manifold perturbations further along the attractor. Experimental design: first we ramp the activity mid-way along the line attractor using activation of *x*_*1*_ neurons, then test the population vector trajectory after targeting of *x*_*2*_ neurons. f. Left: stimulation paradigm. Right: Scheme of the quantification approach for the effect of off manifold targeting further along the attractor. g. State space and the activity ramp following *x*_*1*_ photo-activation (showing only three pulses to avoid clutter). h. Same as Extended Data Fig. 9g but for *x*_*2*_ photo-activation. i. Quantification of the activity distance from baseline after each photostimulation (n = 8 mice, Kruskal-Wallis test with Dunn’s correction for multiple comparison, **p = 0.0025, n.s p > 0.05, error bars - sem). j. Effect of grouped holographic activation of randomly selected neurons on activated neurons. Shaded area – sem, n = 5 mice. k. Average z-score activity of non-targeted *x*_*1*_ dimension upon activation of random neurons. Shaded area – sem n = 5 mice. l. Average z-score activity of non-targeted *x*_*2*_ dimension upon activation of random neurons. Shaded area – sem, n = 5 mice. m. Left: Quantification of activity in non-targeted *x*_*1*_ dimension upon grouped holographic activation of random neurons (n.s, p > 0.05, Kruskal-Wallis test with Dunn’s correction for multiple comparison, n = 5 mice, error bars - sem). Right: Comparison of grouped activation of *x*_*1*_ neurons (green, reproduced from Fig. 2d, right) and grouped activation of random neurons on activity of *x*_*1*_ dimension (black, reproduced from Extended Data Fig. 3m, left, error bars - sem). n. Quantification of activity in non-targeted *x*_*2*_ dimension upon grouped holographic activation of random neurons (n.s, p > 0.05, Kruskal-Wallis test with Dunn’s correction for multiple comparison, n = 5 mice, error bars - sem).

To visualize in neural-state space the effect of reactivating *x*_1_ neurons in the absence of demonstrator mice, we projected the data into a 2D flow-field based on the dynamics matrix fit to data acquired during the observation of aggression. Activation pulses transiently moved the population activity vector (PAV) ‘up’ the line attractor, followed by relaxation back down the attractor to a point that was higher than the initial position of the system (Fig. 2e,f). To quantify this effect, we calculated the Euclidean distance in state space between the initial timepoint during the baseline period (*t*_initial_) and the timepoint at the end of stimulation or at the end of the ISI after each pulse (*t*_stim-end_ and *t*_post-stim_, respectively) (Fig. 2e–h). This revealed that the *x*_1_ perturbations resulted in progressive, stable on-manifold movement along the attractor with each consecutive stimulation, as measured by the increase in both metrics (Fig. 2g,h). However, we found that integration of optogenetic stimulation pulses saturated in the *x*_1_ dimension after the third pulse, suggesting that the line attractor occupies a finite portion of the neural state space (Extended Data Fig. 9a–d).

Importantly, activation of *x*_2_ neurons did not lead to integration (Fig. 2i–k) as predicted by the time constant derived from the fit rSLDS model (Fig. 1e (red bar)). Instead, after each pulse, we observed stimulus-locked transient activity in the *x*_2_ dimension followed by a decay back to the baseline during the ISI period, across stimulation paradigms (Fig. 2k and Extended Data Fig. 8b), with little to no effect on *x*_1_ neurons (Extended Data Fig. 8j–l). In 2D neural-state space, we observed that *x*_2_ neuron activation caused transient off-manifold movements of the PAV orthogonal to the attractor axis during each pulse (Fig. 2l–o). After each stimulus, the PAV relaxed back into the attractor, near to the initial location that it occupied before the stimulus.

To examine further the stability of different points along the line attractor, we performed photostimulation of *x*_2_ neurons after first moving activity in neural-state space further along the attractor using photostimulation of *x*_1_ neurons (Extended Data Fig. 9e, f). This *x*_2_ perturbation also resulted in transient off-manifold movements of the PAV orthogonal to the line attractor, followed by relaxation to the position occupied after the previous *x*_1_ stimulation (but before the *x*_2_ stimulation), rather than simply relaxing back to the baseline (Extended Data Fig. 9g–i). This experiment confirms the attractive nature of different points along the line. Lastly, activation of randomly selected neurons that were not weighted by either dimension did not produce activity along either the *x*_1_ or *x*_2_ dimension, emphasizing the specificity of our on- and off-manifold holographic activation (Extended Data Fig. 9j–n). Activation of either ensemble did not result in overt changes in the behaviour of the head-fixed mouse (Extended Data Fig. 5f–j). Together, these findings demonstrate that a subset of VMHvl^*Esr1*^ neurons (*x*_1_) can integrate direct optogenetic stimulation, moving the PAV along the line attractor, while a different subset (*x*_2_) pushes the PAV out of the attractor.

## Line-attractor neurons form ensembles

**Extended Data Fig. 10:**
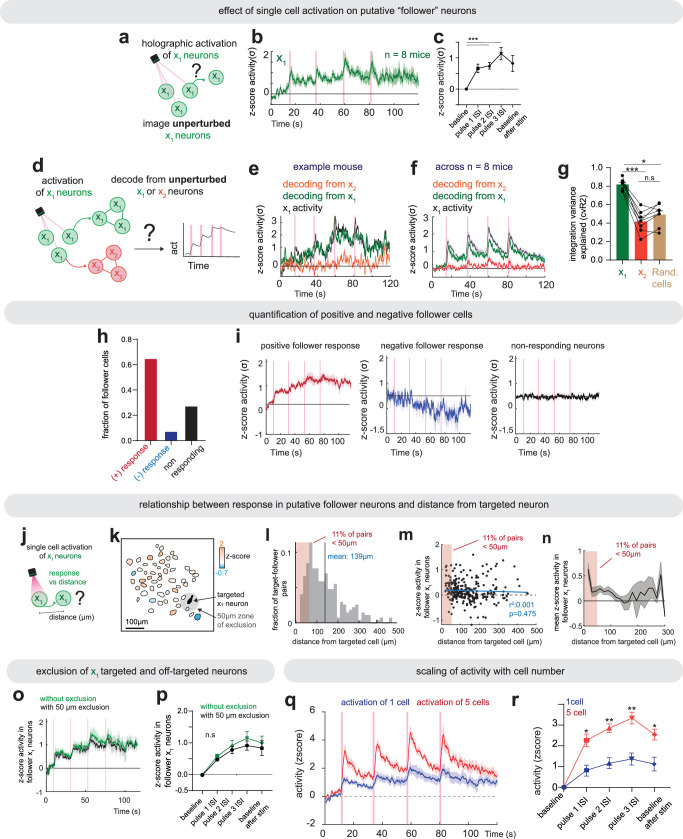
Impact of functional connectivity measurements on non-targeted neurons. a. Experimental design. We grouped activated five *x*_*1*_ neurons (three are shown for illustrative purposes) and examined the activity of non-targeted photoactivated *x*_*1*_ neurons following exclusion of off-target neurons. b. Z-score activity of *x*_*1*_ dimension photoactivated neurons not targeted for photo-stimulation. N = 8 mice. Shaded area – sem. c. Quantification of average z-scored activity of a weighted average of non-targeted *x*_*1*_ dimension neurons during baseline or various inter- photo-stimulation intervals. (n = 8 mice, error bars - sem, ***p<0.001). d. Experimental design for decoding analysis. We examined whether the activity of non-targeted but photoactivated *x*_*1*_ or *x*_*2*_ dimension neurons can be used to decode integration of direct photo-stimulation by groups of five targeted *x*_*1*_ neurons (three are shown for simplicity), using a support vector machine (SVM) decoder. e. One example mouse showing the activity of targeted *x*_*1*_ dimension neurons (black), activity decoded from non-targeted *x*_*1*_ neurons (green), and activity decoded from *x*_*2*_ non-targeted neurons (orange). f. Same as Extended Data Fig. 9e but averaged over 8 mice. Shaded area – sem. g. Decoding from non-targeted *x*_*1*_ neurons can explain significantly more variance (80% versus 40%) than non-targeted *x*_*2*_ or randomly selected neurons (n = 8 mice, *p = 0.01, ***p = 0.0003, n.s. p >0.05, Kruskal Wallis test with Dunn’s correction for multiple comparisons, error bars - sem). h. Fraction of non-targeted neurons with either positive or negative response (defined by whether their mean response post photostimulation of targeted *x*_*1*_ neuron is 1.5 std above or below baseline activity). i. Averaged activity of non-targeted neurons with either a positive (left), negative (middle) or no significant response (right). Shaded area – sem. N = 8 mice. j. Cartoon illustrating how the relationship between spatial distance and response in putative “follower” *x*_*1*_ neurons is assessed. k. Example field of view showing z-score response in all neurons in a field of view. The filled-in black cell is the targeted *x*_*1*_ neuron and the shaded region around it shows a 50 µm stringent zone of exclusion. Putative follower cells are shaded according to their z-score response (see colour scale). Note that some of the most strongly activated cells are located >100 µm from the targeted cell. l. Histogram of distance between targeted *x*_*1*_ neuron and all putative “follower” *x*_*1*_ neurons (mean: 139 ± 35 µm). m. Scatter plot showing the relationship between distance and response in putative “follower” *x*_*1*_ neurons. Blue line shows the regression line. 11% of all assessed putative “follower” *x*_*1*_ neurons are within 50 µm of the targeted *x*_*1*_ neurons. n. Average response from scatter plot in ‘m’. Black line –mean over moving window of 15um. Shaded area – sem. o. Average response in non-targeted *x*_*1*_ neurons from photo-stimulation of single *x*_*1*_ neuron with (black trace) and without (green trace) exclusion of neurons within a 50 µm radius of the targeted neuron (pink shaded region in Extended Data Fig. 10l–n). Shaded area – sem. N = 8 mice. p. Quantification of data from Extended Data Fig. 10o at various time periods after each photo-stimulation pulse. n.s: not significant, Kruskal-Wallis test with Dunn’s correction for multiple comparisons, error bars - sem. N = 8 mice q. *x*_*1*_ integration dimension activity with activation of one neuron (blue) versus five neurons (red). N = 8 mice. Shaded area – sem. r. Quantification of average z-scored activity of projected *x*_*1*_ dimension neurons with one neuron (blue) versus five neurons (red) during baseline or various inter stimulus intervals. N = 8 mice, *p = 0.0239, **p = 0.0063, **p = 0.0074, *p = 0.0341, Kruskal-Wallis test with Dunn’s correction for multiple comparisons, error bars - sem.

The integration observed in the abovementioned experiments could reflect a cell-intrinsic mechanism, or it could emerge from recurrent interactions within a network^40^. To determine whether the latter mechanism contributes to the line attractor, we first examined whether putative *x*_1_ follower cells (that is, non-targeted neurons that were photoactivated by stimulation of targeted *x*_1_ neurons) exhibited integration. Indeed, even after excluding the targeted *x*_1_ neurons themselves as well as potentially off-target neurons located within a 50 µm radius of the targeted cell (Extended Data Figs. 6a–h and 10j–n), we observed integration in the remaining *x*_1_ neurons (Extended Data Fig. 10a–c). Moreover, optogenetically evoked integrated activity in targeted *x*_1_ neurons could be reliably decoded from the activity of their follower *x*_1_ neurons (Extended Data Fig. 10d–f). This decoding was significantly better than that obtained using the activity of non-targeted *x*_2_ neurons; furthermore, the *x*_2_ activity-based decoder performance was slightly worse than decoders trained on neurons chosen randomly (Extended Data Fig. 10g). These analyses suggest that selective functional connectivity between integration dimension-weighted *x*_1_ neurons contributes to line-attractor dynamics in the VMHvl.

**Fig. 3:**
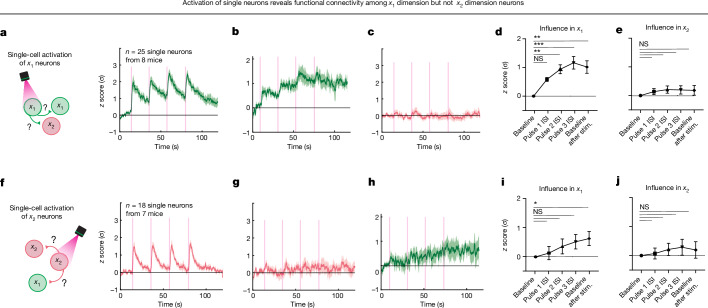
Neural implementation of a line attractor by functional connectivity. **a**, Left, the paradigm for examining activity in non-targeted *x*_1_ and *x*_2_ neurons after activation of unitary *x*_1_ neurons. Right, the average *z*-scored activity of the perturbed (targeted) *x*_1_ neurons (25 single neurons from *n* = 8 mice). Data are the average trace (dark green) ± s.e.m. (shaded green area). **b**, The average *z*-scored activity of non-targeted *x*_1_ neurons after targeting unitary *x*_1_ neurons. *n* = 8 mice. Data are average trace (dark green) ± s.e.m. (shaded green area). **c**, The average *z*-scored activity of non-targeted *x*_2_ neurons after targeting of unitary *x*_1_ neurons. *n* = 8 mice. Data are average (trace in dark red) ± s.e.m. (shaded red area). **d**, Quantification of activity in non-targeted *x*_1_ neurons after targeting of single *x*_1_ neurons. NS, *P* = 0.16; **P = 0.0037 (bottom), ***P = 0.0005, **P = 0.0016 (top). *n* = 8 mice. Data are mean ± s.e.m. **e**, Quantification of the activity in non-targeted *x*_2_ neurons after targeting of single *x*_1_ neurons (NS; *n* = 8 mice). Data are mean ± s.e.m. **f**, The paradigm for examining the activity in non-targeted *x*_1_ and *x*_2_ neurons after activation of single *x*_2_ neurons (left). Right, the average *z*-scored activity of targeted *x*_2_ neurons (18 single neurons from *n* = 7 mice). Data are the average trace (dark red) ± s.e.m. (shaded red area). **g**, The average *z*-scored activity of non-targeted *x*_2_ neurons after targeting of single *x*_2_ neurons. *n* = 7 mice. Data are the average trace (dark red) ± s.e.m. (shaded red area). **h**, The average *z*-scored activity of non-targeted *x*_1_ neurons after targeting of single *x*_2_ neurons. *n* = 7 mice. Data are the average trace (dark green) ± s.e.m. (shaded green area). **i**, Quantification of activity in non-targeted *x*_1_ neurons after targeting of single *x*_2_ neurons. NS, from bottom to top, *P* = 0.999, *P* = 0.31, *P* = 0.09; *P = 0.0316. *n* = 7 mice. Data are mean ± s.e.m. **j**, Quantification of activity in non-targeted *x*_2_ neurons after targeting of single *x*_2_ neurons (NS). n = 7 mice. Data are mean ± s.e.m.

To assess more precisely the extent of functional connectivity among VMHvl^*Esr1*^ neurons, we activated unitary *x*_1_ or *x*_2_ neurons and performed imaging of non-targeted neurons (Fig. 3a). These experiments revealed a slowly decaying elevation of activity during the ISI period in non-targeted *x*_1_ neurons after each pulse of activation (Fig. 3b,d) that was mostly positive (Extended Data Fig. 10h,i). Notably, the strength of functional connectivity was not positively correlated with the distance from the targeted photostimulated cell (Extended Data Fig. 10j–n) and was still observed even after excluding neurons in a 50 µm zone surrounding the targeted neuron to eliminate potential off-target effects due to ‘spillover’ photostimulation (Extended Data Fig. 10o,p). Comparing the activity of non-targeted photoactivated *x*_1_ neurons during unitary *x*_1_ neuron photoactivation versus during targeted activation of the five-*x*_1_ neuron cohorts revealed that the response strength of the non-targeted *x*_1_ neurons scaled with the number of targeted *x*_1_ neurons (Extended Data Fig. 10q,r). Importantly, the observed functional coupling between *x*_1_ neurons could not be explained by local clustering of non-targeted *x*_1_ neurons near the targeted cell (Extended Data Figs. 6i–k and 10k–l).

In contrast to the observed *x*_1_-to-*x*_1_ functional connectivity, we observed little activity in non-targeted *x*_2_ neurons after activation of unitary *x*_1_ or *x*_2_ neurons (Fig. 3c,e,g,j), suggesting that functional *x*_1_–*x*_1_ connectivity is selective. While there was a trend to a gradual increase in activity in non-targeted *x*_1_ neurons after repeated activation of unitary *x*_2_ neurons (Fig. 3f–h), that increase was not statistically significant (Fig. 3i,j).

**Fig. 4:**
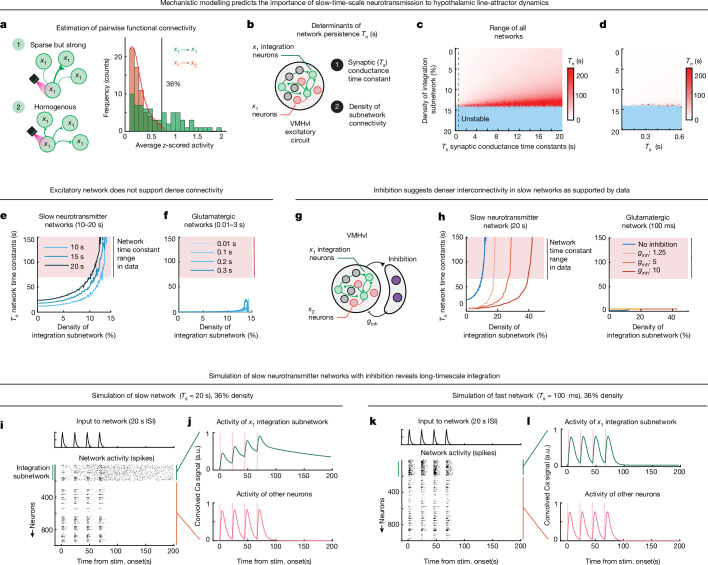
Mechanistic modelling suggests slow neurotransmission and feedback inhibition. **a**, Diagram of strong but sparse connectivity among *x*_1_ neurons (1), or dense interconnectivity within subnetwork (2) (left). Right, the empirical distribution of the strength of pairwise functional connectivity between *x*_1_ neurons (green) and from *x*_1_ to *x*_2_ neurons (red). *n* = 99 pairs, *n* = 7 mice. **b**, Cartoon illustrating different elements of an excitatory network that can determine network-level persistent activity. **c**, Model simulation result showing the network time constant (*τ*_n_) by varying the subnetwork connectivity (*σ*) in the range of 0 to 20% density values and *τ*_s_ in the range of 0 to 20 s. Blue portions show configurations that result in unstable networks with runaway excitation. **d**, Magnified version of **c** (the region left of the dashed line) showing glutamatergic networks with a synaptic conductance time constant (*τ*_s_) in range of 0.01 to 0.6 s. **e**, Network time constant (*τ*_n_) against density of integration subnetwork for slow neurotransmitter (*τ*_s_: 10, 15 and 20 s). *τ*_n_ varies monotonically with density for large values of *τ*_s_. **f**, As in **e** but for glutamatergic networks (*τ*_s_: 0.01, 0.1, 0.2 and 0.3 s). **g**, Cartoon showing the modified VMHvl circuit with fast feedback inhibition incorporated. **h**, Plot of network time constant (*τ*_n_) against density of integration subnetwork for a slow neurotransmitter network with *τ*_s_ = 20s, for different values of strength of inhibition (inhibitory gain, *g*_inh_: 1.25, 5 and 10) (left). Right, as on the left but for a glutamatergic network with *t*_s_ = 0.1 s. **i**, Model simulation of a slow neurotransmitter network with fast feedback inhibition (*t*_s_: 20 s, 36% density of subnetwork connectivity). Top, the input (20 s ISI) provided to the model, Bottom, spiking activity in the network. The first 200 neurons (20%) comprise the interconnected integration subnetwork. **j**, Ca^2+^ activity convolved from firing rate (Methods) of the integration subnetwork (top) and the remaining neurons (bottom). **k**, As in **i** but for a fast transmitter network (*t*_s_: 0.1 s, 36% density of subnetwork connectivity). **l**, As in **j** but for a fast transmitter network (*t*_s_: 0.1 s, 36% density of subnetwork connectivity). a.u., arbitrary units.

The functional connectivity that we observed could arise either from a population of sparsely but strongly interconnected neurons, or from a population with denser connections of intermediate strength^41^ (Fig. 4a (left)). To assess this, we calculated the distribution of pairwise influence scores in our unitary neuron stimulation experiments, defined as the average evoked *z*-scored activity in each non-targeted photoactivated *x*_1_ neuron after photostimulation of a single targeted cell. To estimate the amount of functional coupling within the *x*_1_ network, we considered the percentage of *x*_1_→*x*_1_ pairs that had influence scores higher than the highest *x*_1_→*x*_2_ pair, which had a *z* score of around 0.6 (Fig. 4a (right, vertical line)). The fraction of *x*_1_→*x*_1_ pairs above this threshold was around 36% (Fig. 4a (right)). These data suggest that VMHvl^*Esr1*^ neurons that contribute to the line attractor form relatively dense functional ensembles, consistent with theory-based predictions^40^.

**Extended Data Fig. 11:**
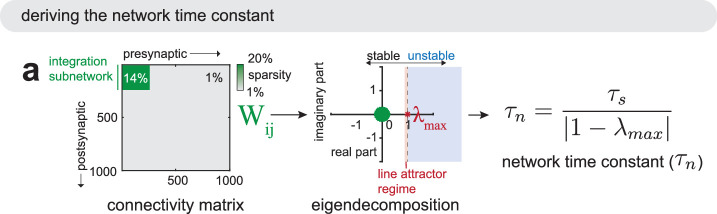
Deriving network time constant for model simulations. a. Analytical derivation of network time constant from connectivity matrix of purely excitatory recurrent neural network.

We next used computational approaches to investigate the kinetics of the observed functional connectivity within *x*_1_ ensembles. Such connectivity could reflect either fast, glutamatergic synapses, as typically assumed for most attractor networks^40^; or they could be slow neuromodulator-based connections that use GPCR-mediated second messenger pathways to sustain long-time-scale changes in synaptic conductance. To investigate systematically the density and synaptic kinetics of networks capable of generating line attractorlike dynamics with the measured integration-dimension (*x*_1_) network time constants, we turned to mechanistic modelling using an excitatory integrate and fire network^7^ (Fig. 4b). As VMHvl is >80% glutamatergic^42^, we used excitatory networks and analytically calculated the network time constant using an eigen-decomposition of the connectivity matrix^40^ (Extended Data Fig. 11a). By varying the synaptic conductance time constant (*τ*_s_) and the density of the integration subnetwork connectivity, we found that only artificial networks based on relatively sparse connectivity (around 8–12%) and slow synaptic time constants (around 20 s) could yield network time constants (*τ*_n_) in the experimentally observed range (~50–200 s; Fig. 4c,e (red shading)). By contrast, networks with fast glutamatergic connectivity failed to do so over the same range of connection densities (Fig. 4d,f).

In these purely excitatory network models, the density of connections that yielded network time constants in the observed range was much lower than the experimentally measured value (36%). To match more accurately the empirically observed connection density, we incorporated excitation-recruited fast-feedback inhibition into our integrate-and-fire network^7^, as VMHvl is known to receive dense GABAergic innervation from surrounding areas^43,44^. The addition of global strong feedback inhibition allowed networks to match the observed connection density (36%) but, importantly, maintained the slow nature of the functional connectivity (20 s; Fig. 4g and 4h (left)). Indeed, networks simulated with a long *τ*_s_ (20s) and dense *σ* (36%) could integrate digital optogenetic stimulation in a manner like that observed experimentally (Fig. 4i,j). By contrast, purely glutamatergic networks (*τ*_s_ = 100 ms) were unable to integrate at the observed timescales given the measured connectivity density (Fig. 4h (right) and 4k–l). Together, these results suggest an implementation of the VMHvl^*Esr1*^ line attractor that combines slow neurotransmission and relatively dense^41^ subnetwork interconnectivity within an attractor-creating ensemble.

## Attractor stability ties to connectivity

**Fig. 5:**
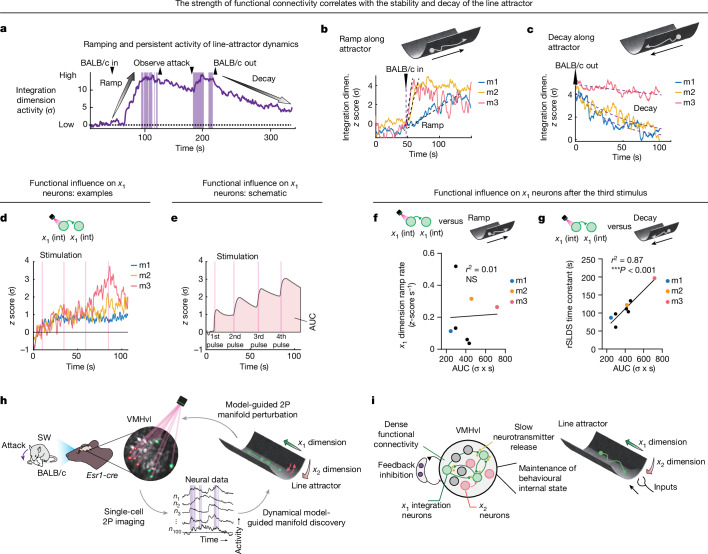
The strength of functional connectivity reflects line-attractor stability. **a**, Example neural activity projected onto the *x*_1_ (integration) dimension (dimen.) of one mouse observing aggression, demonstrating a ramp when the BALB/c intruder enters the demonstrator cage containing an aggressive SW mouse (that is, movement up the line attractor) and decay after removal of the BALB/c intruder from the demonstrator cage (that is, movement down the line attractor). **b**, Dynamics of the integration dimension aligned to the entry of the BALB/c intruder for three example mice. Note the different rates of ramping in different mice. Norm. act., normalized activity. **c**, As in **b**, aligned to the removal of the BALB/c intruder, showing different rates of decay. **d**, *z*-scored activity of non-targeted *x*_1_ neurons after activation of individual *x*_1_ neurons in mice from **b** and **c**. The pink vertical lines show photostimulation pulses. **e**, Illustration of different quantitative metrics of the change in activity of non-targeted *x*_1_ neurons from **d** as either the average *z*-scored activity, or the area under the curve (AUC). The pink vertical lines show the photostimulation pulses. **f**, Correlation between the rate of ramping of the integration dimension from the rSLDS model fit to data obtained from observation of aggression, and the AUC of non-targeted *x*_1_ neurons measured using AUC after the third stimulus (*r*^2^ = 0.01; NS). *n* = 8 mice. **g**, The correlation between the rSLDS time constant (decay rate of the integration dimension) obtained from observation of aggression, and the AUC of non-targeted *x*_1_ neurons measured after the third stimulus (*R*^2^ = 0.87; ***P < 0.001). *n* = 8 mice. **h**, Summary of the results illustrating causal evidence of a hypothalamic line attractor. **i**, Diagram of the implementation of a hypothalamic line attractor encoding a behavioural internal state.

**Extended Data Fig. 12:**
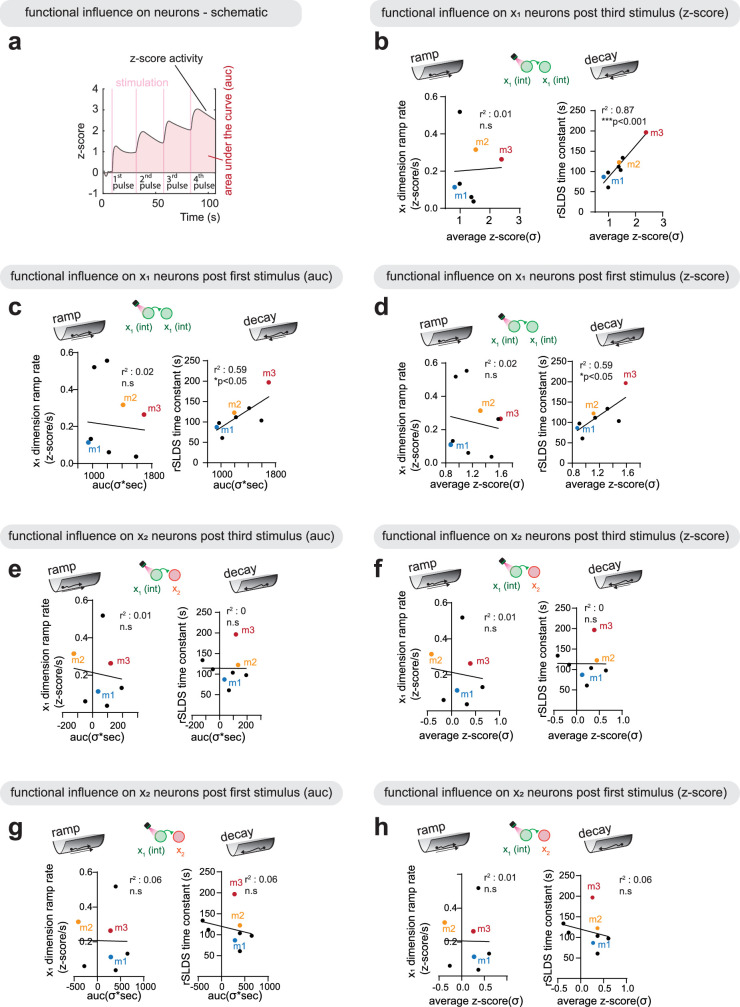
Additional quantifications of the correlation between functional connectivity and the stability of the decay and ramp. a. Illustration of different quantification approaches to the change in activity of non-targeted *x*_*1*_ neurons from Fig. 5e as either the average z-score activity following different stimulus pulses, or the area under the curve (auc). Red vertical lines, photostimulation pulses. b. Left: Correlation between the rate of ramping of the integration dimension obtained from observation of aggression and average z-score of non-targeted *x*_*1*_ neurons measured using the average z-score post third stimulus (r^2^: 0.01, n.s, n = 8 mice). Right: Correlation between rSLDS time constant obtained from observation of aggression and average z-score across non-targeted *x*_*2*_ neurons measured using the average z-score post third stimulus (r^2^: 0.87, ***p < 0.001, n = 8 mice). c. Same as b) but calculated from non-targeted *x*_*1*_ neurons measuring the auc of activity post first stimulus. d. Same as c), calculated from non-targeted *x*_*1*_ neurons measuring the average z-score activity. e. Same as c) but calculated from non-targeted *x*_*2*_ neurons measuring the AUC of activity post third stimulus. f. Same as e) but calculated using the average z-score activity. g. Same as e) but calculated post first stimulus. h. Same as g) but calculated using the average z-score activity.

The observed dynamics along the integration dimension exhibits two important characteristics that can reflect the stability of the line attractor, ramping activity up; and slow decay down the integrator (Fig. 5a). Both of these characteristics might either be intrinsic or be driven by external inputs to the line attractor^5,40^. Previously, we observed that individual differences in aggressiveness among mice were positively correlated with the stability and decay of the VMHvl line attractor during aggression^5^. We therefore investigated whether individual differences in line-attractor ramping or rate of decay might also be correlated with the strength of functional connectivity within the *x*_1_ ensemble (Fig. 5b–d). We plotted either the *x*_1_ decay time constants, or the rate of ramp up along the *x*_1_ dimension (obtained from rSLDS models fit to each mouse using data recorded during attack observation), against different quantitative metrics of functional connectivity between targeted *x*_1_ or *x*_2_ neurons and their non-targeted putative follower cells (obtained from the same animals by single-cell optogenetic stimulation and imaging after removal of the demonstrator intruder mice) (Fig. 5d,e and Extended Data Fig. 12a).

Notably, there was a strong correlation across mice between the time constant (decay) of the line attractor measured during the observation of aggression, and the strength of functional connectivity among integration-dimension (*x*_1_) neurons measured by post-observation optogenetic stimulation (Extended Data Fig. 12c,d). The strength of this correlation was higher after the third stimulus (*r*^2^ = 0.87) compared with after the first stimulus (*r*^2^ = 0.59) (Fig. 5g and Extended Data Fig. 12b), indicating that individual differences in the attractor time constant become more apparent once the system has already integrated several inputs, thereby taking longer to decay. By contrast, there was no correlation between functional connectivity and the rate of ramp-up, suggesting that the latter might be driven by extrinsic inputs to the VMHvl (Fig. 5f and Extended Data Fig. 12b–d). Importantly, the correlation between attractor stability and functional connectivity was specific to neurons in the integration (*x*_1_) subnetwork, and did not hold when rSLDS time constants were compared with the influence strength of targeted *x*_1_ neurons on *x*_2_ cells (Extended Data Fig. 12e–h). Thus, individual differences among mice in the stability of the line attractor during the observation of aggression are correlated with individual differences in the functional connection strength among attractor-contributing neurons.

## Discussion

Using model-guided closed-loop all-optical experiments, we provide causal evidence of line attractor-like dynamics in a mammalian system (Fig. 5h,i). Our data and modelling also provide insights into the implementation of the approximate line attractor^5^. We found evidence of relatively dense, selective connectivity among a physiologically distinct subset of *Esr1*^*+*^ neurons. Whether this subset corresponds to one of the transcriptomically distinct subtypes of *Esr1*^*+*^ neurons remains to be determined^31^. Our models confirm the importance of rapid feedback inhibition^7^, consistent with studies of the *Drosophila* ring attractor^21,45^. However they differ from most continuous attractor models^3,40^ by invoking slow neuromodulatory transmission rather than fast glutamatergic excitation. Numerous theoretical studies have posited that continuous attractors relying on recurrent glutamatergic connectivity require precise tuning of synaptic weights to sustain stable attractor dynamics^40,46,47^. By contrast, the inclusion of slow neurotransmission in our mechanistic models yielded network time constants in the observed range across a wide range of connectivity densities. This slow neurotransmission may have evolved not only to ensure attractor robustness, but also to implement the relatively long time scales of internal affective or motive states. These slow dynamics could be implemented by GPCR-mediated signalling triggered by biogenic amines or neuropeptides^48^. Consistent with this prediction, we have recently found that VMHvl line-attractor dynamics and aggression are dependent on signalling through oxytocin and/or vasopressin neuropeptide receptors expressed in *Esr1*^*+*^ neurons^49^. However, that does not exclude a contribution from recurrent glutamatergic excitation in the ventromedial hypothalamus, as in line attractors that mediate cognitive functions on shorter time scales^14,50^.

Lastly, our observations indicate a pronounced correlation between individual differences in the functional strength of integration subnetwork connectivity and differences in the measured stability of the line attractor, perhaps reflecting a leaky integrator. We previously found that, in freely behaving animals, individual differences in attractor stability were correlated with individual differences in aggressiveness^5^. By transitivity, this suggests that individual differences in the strength of functional connectivity within the attractor network might underlie individual differences in aggressiveness. As these differences are observed among genetically identical inbred mice, these observations suggest that attributes of the attractor, such as its connectivity density or strength, may be modifiable (either by epigenetic mechanisms and/or experience^26^). Deciphering the underlying mechanisms that afford this attractor its apparent flexibility while maintaining its robustness represents a promising avenue for future research.

## Methods

### Mice

All of the experimental procedures involving the use of live mice, or their tissues were carried out in accordance with NIH guidelines and were approved by the Institute Animal Care and Use Committee and the Institute Biosafety Committee at the California Institute of Technology (Caltech). All C57BL/6N mice used in this study, including wild-type and transgenic mice, were bred at Caltech. Swiss Webster (SW) male residents and BALB/c male intruder mice were bred at Caltech. Experimental C57BL/6N mice and resident SW mice were used at the age of 8–20 weeks. Intruder BALB/c mice were used at the age of 6–12 weeks and were maintained with three to five cage mates to reduce their aggression. *Esr1*^*cre*/+^ knock-in mice (Jackson Laboratory, 017911) were back-crossed into the C57BL/6N background and bred at Caltech. Heterozygous *Esr1*^*cre/+*^ mice were used for cell-specific targeting experiments and were genotyped by PCR analysis using genomic DNA from ear tissue. All mice were housed in ventilated microisolator cages in a temperature-controlled environment (median temperature, 23 °C, humidity, 60%), under a reversed 11 h–13 h dark–light cycle, with ad libitum access to food and water. Mouse cages were changed weekly.

### Viruses

The following adeno-associated viruses (AAVs), along with the supplier, injection titres and injection volumes, were used in this study: AAV1-syn-FLEX-jGCaMP7s-WPRE (Addgene, 104492, around 2 × 10^12^ viral genomes per ml, 200 nl per injection), AAVdj-Ef1a-DIO-ChRmine-mScarlet-Kv2.1-WPRE (Janelia Vector Core, around 2 × 10^12^ viral genomes per ml, 200 nl per injection).

### Histology

After completion of 2P/miniscope experiments, histological verification of virus expression and implant placement were performed on all of the mice. Mice lacking virus expression or correct implant placement were excluded from the analysis. Mice were perfused transcardially with 0.9% saline at room temperature, followed by 4% paraformaldehyde in 1× PBS. Brains were extracted and post-fixed in 4% paraformaldehyde overnight at 4 °C, followed by 24 h in 30% sucrose/PBS at 4 °C. The brains were embedded in OCT mounting medium, frozen on dry ice and stored at −80 °C for subsequent sectioning. Brains were sectioned at a thickness of 80 μm on a cryostat (Leica Biosystems). The sections were washed with 1× PBS and mounted onto Superfrost slides, then incubated for 30 min at room temperature in DAPI/PBS (0.5 μg ml^−1^) for counterstaining, washed again and a cover slip was added. The sections were imaged with epifluorescence microscope (Olympus, VS120).

### Stereotaxic surgeries

Surgeries were performed on sexually experienced adult male *Esr1*^*cre/+*^ mice aged 6–12 weeks. Virus injection and implantation were performed as described previously^25,51^. In brief, mice were anaesthetized with isoflurane (5% for induction and 1.5% for maintenance) and placed onto a stereotaxic frame (David Kopf Instruments). Virus was injected into the target area using a pulled-glass capillary (World Precision Instruments) and a pressure injector (Micro4 controller, World Precision Instruments), at a flow rate of 50 nl min^−1^. The glass capillary was left in place for 5 min after injection before withdrawal. Stereotaxic injection coordinates were based on the Paxinos and Franklin atlas^52^. Virus injection: VMHvl, anteroposterior (AP), −1.5; mediolateral (ML), ±0.75; dorsoventral (DV), −5.75. For 2P experiments GRIN lenses (0.6 × 7.3 mm, Inscopix) were slowly lowered into the brain and fixed to the skull with dental cement (Metabond, Parkell). Coordinates for GRIN lens implantation: VMHvl, AP, −1.5; ML, −0.75; DV, −5.55). A permanent head bar was attached to the skull with Secure Resin cement (Parkell). For microendoscopy experiments, an additional baseplate was attached to the skull (Inscopix).

### Housing conditions for behavioural experiments

All male C57BL/6N mice used in this study were socially and sexually experienced. Mice aged 8–12 weeks were initially co-housed with a female C57BL/6N female mouse for 1 day and were then screened for attack behaviours. Mice that showed attack towards males during a 10 min resident intruder assay were selected for surgery and subsequent behaviour experiments. From this point forward, these male mice were always co-housed with a female.

### Behaviour annotations

Behaviour videos were manually annotated using a custom MATLAB-based behaviour annotation interface^53,54^. A ‘baseline’ period of 5 min when the animal was alone in its home cage was recorded at the start of every recording session. Two behaviours during the resident intruder assays were annotated: sniff (face, body, genital-directed sniffing) towards male intruders, and attack (bite, lunge).

### Behavioural assays

An observation arena was built from a transparent acrylic (18 × 12.5 × 18 cm, length × width × height), and a perforated part was put in front of the mice observing aggression. Perforations were 1.27 cm diameter and spread evenly throughout the bottom third of the panel. Before initiation of the assay, the observation arena was scattered with soiled bedding from the cage of the aggressive SW demonstrator. For observation of aggression in freely behaving animals (miniscope experiments), an observer was first habituated for 15 min. A singly housed SW male demonstrator was then introduced into the observation arena, followed 1 min later with the insertion of a socially housed stimulus male (BALB/c) mouse into the same compartment. The observation of aggressive encounters persisted for around 1 min, then, after 2 min, a different intruder was introduced for another minute. Observation assays were conducted under white-light illumination. For experiments in engaging aggression, the resident mouse was first habituated 15 min then a BALB/c intruder mouse was introduced twice for 1–2 min. For the experiments comparing neural activity of mice observing aggression and mice engaging aggression, we randomly changed the order of sessions. For mice observing aggression in the 2P set-up, the approach was similar, except that the observer mouse was head-fixed and on a treadmill instead of freely behaving in his home cage.

### Microendoscopy imaging

On the day of imaging, the mice were habituated for at least 15 min after installation of the miniscope in their home cage before the start of the behaviour tests. Imaging data were acquired at 30 Hz with 2× spatial downsampling; light-emitting diode power (0.1–0.5) and gain (1–7×) were adjusted depending on the brightness of GCaMP expression as determined by the image histogram according to the user manual. A transistor–transistor logic pulse from the Sync port of the data acquisition box (Inscopix) was used for synchronous triggering of StreamPix7 (Norpix) for video recording.

### 2P imaging and holographic optogenetics

Two to three weeks after surgery, the mice were habituated to the experimenter’s hand by handling for 15 min a day for three consecutive days. Once the mice were habituated to the experimenter’s hand, they were manually scooped and gently placed onto the treadmill. Mice were head-fixed for 3 consecutive days for habituation. Head fixation was achieved by securing the head bar into a metal clamp attached to a custom head stage. During habituation, the mice were placed underneath the objective for 15 min and given access to random presentations of chocolate milk. After habituation, combined 2P imaging and behaviour sessions were conducted. jGCaMP7s imaging was acquired using an Ultima 2P Plus and the Prairie View Software (Bruker Fluorescence Microscopy). Individual frames were acquired at 10 Hz using a galvo-resonant scanner with a resolution of 1,024 px × 1,024 px. We used a long-working-distance ×20 air objective designed for infrared wavelengths (Olympus, LCPLN20XIR, 0.45 NA, 8.3 mm working distance) combined with a femtosecond-pulsed laser beam (Chameleon Discovery, Coherent). GCaMP was excited using a 920 nm wavelength. For targeted photostimulation, the same microscope and acquisition system (Bruker) was used with a second laser path consisting of a 1,035 nm high power femtosecond pulsed laser (Monaco 1035-40-40, Coherent), spatial light modulator (512 × 512 px density) to generate multipoint stimulation montages (NeuraLight 3D, Bruker). During photostimulation, the mice were head-fixed in complete darkness on a rotating cylinder that enabled them to run. Neurons were selected for targeted photostimulation based on two criteria: (1) their weights from the rSLDS model and (2) if they responded to photostimulation. In case a neuron did not show a significant increase in activity in response to photostimulation, a new neuron was chosen until a total of five photosensitive neurons was targeted for each grouped stimulation experiment (Fig. 2). During the photostimulation session, a 128-frame average image was generated to clearly highlight all neurons. To reduce off-target effects during photostimulation, we used small targets (10 µm diameter) that were manually restricted to GCaMP-expressing neurons. Moreover, the laser power was adjusted to be a maximum of 5 mW per target. We used Prairie software to elicit holographic photostimulation (10 Hz, 2 s, 10 ms pulse width). Photostimulations were done between frames to avoid laser artefacts. Importantly, to reduce cross activation of the ChRmine from the 920 nm laser, we kept laser power for imaging to be less than 30 mW.

To extract regions of interest, data from mice observing aggression was uploaded to ImageJ. Videos were then motion corrected using the moco plugin^55^. Motion-corrected videos were averaged, and additional contrast and brightness adjustments were made to clearly highlight all neurons in the FOV. Cells were then manually extracted and an rSLDS model was used to identify *x*_1_ and *x*_2_ dimension neurons. Neurons were then identified on the FOV using the Prairie view software and were targeted for photostimulation. While rSLDS models was running (15–20 min, see below), control experiments were conducted.

### Microendoscopy data extraction

#### Preprocessing

Miniscope data were acquired using the Inscopix Data Acquisition Software as 2× downsampled .isxd files. Preprocessing and motion correction were performed using Inscopix Data Processing Software. In brief, raw imaging data were cropped, 2× downsampled, median filtered and motion corrected. A spatial band-pass filter was then applied to remove out-of-focus background. Filtered imaging data were temporally downsampled to 10 Hz and exported as a .tiff image stack.

#### Calcium data extraction

After preprocessing, calcium traces were extracted and deconvolved using the CNMF-E^56^ large data pipeline with the following parameters: patch_dims = [4], gSig = 3, gSiz = 13, ring_radius = 17, min_corr = 0.7, min_pnr = 8. The spatial and temporal components of every extracted unit were carefully inspected manually (SNR, PNR, size, motion artefacts, decay kinetics and so on) and outliers (obvious deviations from the normal distribution) were discarded.

#### Terminology

We use the following terminology to refer to the design and results of our experiments:

1. *x*_1_ or *x*_2_ neurons: cells that were identified by rSLDS modelling as contributing to dimensions *x*_1_ or *x*_2_, respectively, during observation of aggression.
2. Targeted neurons: rSLDS-identified cells that were purposely photostimulated.
3. Photoactivated neurons: cells that were empirically found to increase their ∆*F*/*F* in response to photostimulation of one or more targeted neurons, that is, photoresponsive neurons. This category includes both the purposely stimulated (targeted) and not purposely stimulated neurons. The latter may include both off-target neurons and putative follower cells.
4. Off-target neurons: photoactivated neurons that were not purposely photostimulated, but which responded to photostimulation of a selected target cell(s) with an increased ∆*F*/*F* because they were close enough (within 15 µm) to be inadvertently activated by light spillover from the targeted neuron (Extended Data Fig. 6a–h).
5. Putative follower cells: neurons that responded to photostimulation and that were outside a 50 µm radius around the targeted cell (to conservatively exclude off-target neurons; Extended Data Figs. 6h and 10k–n); they are putative targets (direct or indirect) of the targeted cell.

#### Dynamical system models of neural data

rSLDS models^16,29^ were fit to neural data as previously described^15^. In brief, rSLDS is a generative state-space model that decomposes nonlinear timeseries data into a set of discrete states, each with simple linear dynamics. The model describes three sets of variables: a set of discrete states (*z*), a set of latent factors (*x*) that captures the low-dimensional nature of neural activity and the activity of recorded neurons (*y*). While the model can also allow for the incorporation of external inputs based on behaviour features, such external inputs were not included in our first analysis.

The model is formulated as follows: at each timepoint, there is a discrete state *z*_*t*_ ∈ {1, …, *K*} that depends recurrently on the continuous latent factors (*x*) as follows:

1 $$p\left({z}_{t+1}{\rm{| }}{z}_{t}=k,{x}_{t}\right)={\rm{softmax}}\left\{{R}_{k}{x}_{t}+{r}_{k}\right\}$$

where ${R}_{k}\in {{\mathbb{R}}}^{K\times K}$ and ${r}_{k}\in {{\mathbb{R}}}^{K}$ parameterize a map from the previous discrete state and continuous state to a distribution over the next discrete states using a softmax link function. The discrete state *z*_*t*_ determines the linear dynamical system used to generate the latent factors at any time *t*:

2 $${x}_{t}={A}_{{z}_{t}}{x}_{t-1}+{b}_{{z}_{t}}+{{\epsilon }}_{t}$$

where ${A}_{k}\in {{\mathbb{R}}}^{d\times d}$ is a dynamics matrix and ${b}_{k}\in {{\mathbb{R}}}^{D}$ is a bias vector, where *D* is the dimensionality of the latent space and ${{\epsilon }}_{t}\, \sim {N}(0,{Q}_{{z}_{t}})$ is a Gaussian-distributed noise (also known as innovation) term.

Lastly, we can recover the activity of recorded neurons by modelling activity as a linear noisy Gaussian observation ${y}_{t}\,\in {{\mathbb{R}}}^{N}$ where *N* is the number of recorded neurons:

3 $${y}_{t}=C{x}_{t}+d+{\delta }_{t}$$

For $C\in {{\mathbb{R}}}^{N\times D}$ and ${\delta }_{t} \sim N(0,S)$, a Gaussian noise term. Overall, the system parameters that rSLDS needs to learn consists of the state transition dynamics, library of linear dynamical system matrices and neuron-specific emission parameters, which we write as:

4 $$\theta =\{{\{{A}_{k}{b}_{k},\,{Q}_{k},\,{R}_{k},\,{r}_{k}\}}_{k=1}^{K},\,C,\,d,\,S\}$$

We evaluate model performance using both the evidence lower bound and the forward simulation accuracy (Fig. 3a) described previously^5,15^, as well as by calculating the variance explained by the model on data.

We used two-dimensional models, selecting the optimal number of states through fivefold cross-validation. To ascertain which neurons contributed to each of the two model dimensions (*x*_1_ and *x*_2_), we initially confirmed the orthogonality of these dimensions by computing the subspace angle between them (88.1 ± 0.87°, *n* = 9 mice). Given this near orthogonality, we then used the columns of the emission matrix *C* to identify neurons that contributed to the two separate dimensions of the model.

The contribution of neurons to each latent dimension is defined based on their weights from the emission matrix *C*, which is initialized by factor analysis and then optimized by rSLDS. In the matrix *C*, the rows define the weights that create the latent dimensions and the columns defined the different latent dimensions (*x*_1_ and *x*_2_) in the model. The model performance is reported both as the evidence lower bound, which is equivalent to the Kullback–Leibler divergence between the approximate and true posterior as well as the variance (cross-validated *R*^2^ (cvR^2^)) explained. We cross-validated the model using fivefold cross-validation, for which we trained the data on four arbitrary portions of the data and tested on a left out fifth portion. In all of the experiments, the model must achieve at least 70% cvR^2^ before it is used for downstream analysis such as identification of *x*_1_ and *x*_2_ neurons. Models fit to miniscope data during engagement of aggression obtained a cvR^2^ = 84.7 ± 0.03%, while the same model explains 67.2 ± 0.02% of variance in data obtained from observation of aggression. Flow fields obtained from head-fixed animals observing aggression where fit with input terms representing the presence of the BALB/c intruder.

#### Estimation of time constants

We estimated the time constant of each dimension of linear dynamical systems using eigenvalues *λ*_*a*_ of the dynamics matrix of that system, derived previously as^57^:

5 $${\tau }_{a}=\left|\frac{1}{\log (\left|{\lambda }_{a}\right|)}\right|$$

The intrinsic leak rate is defined based on the time constant of the integration dimension across the whole session. The activity observed by the model takes into account both decays (that is, the decays after the first and second time the intruder is removed), and therefore gives high prediction to the holographic perturbation experiments (cVR^2^, ~85%; Fig. 2f,p). Note also that the dynamics captured by the perturbation experiments more closely resembles the second intruder interaction rather than the first. Furthermore, the SW mouse is still in the observation chamber between BALB/c intruders, but is removed after the second intruder. For this reason, the observed dynamics is mostly consistent and across mice the second decay seems faster.

#### Calculation of line attractor score

To provide a quantitative measure of the presence of line-attractor dynamics, we devised a line attractor score as defined previously^5^ as:

6 $${\rm{line}}\;{\rm{attractor}}\;{\rm{score}}={\log }_{2}\frac{{t}_{n}}{{t}_{n-1}}$$

where *t*_*n*_ is the largest time constant of the dynamics matrix of a dynamical system and ${t}_{n-1}$ is the second largest time constant.

#### Calculation of autocorrelation half-width

We computed autocorrelation halfwidths by calculating the autocorrelation function for each neuron timeseries data (*y*_*t*_) for a set of lags as described previously^12^. In brief, for a time series (*y*_*t*_), the autocorrelation for lag *k* is:

7 $${r}_{k}=\frac{{c}_{k}}{{c}_{0}}$$

where *c*_*k*_ is defined as:

8 $${c}_{k}=\frac{1}{T}\mathop{\sum }\limits_{t=1}^{T-k}({y}_{t}-\bar{y})({y}_{t+k}-\bar{y})$$

and *c*_*0*_ is the sample variance of the data.

#### Mechanistic modelling

We constructed a model population of *n* = 1,000 standard current-based leaky integrate-and-fire neurons as previously performed^7^. We first modelled a purely excitatory spiking network in which each neuron has membrane potential *x*_*i*_ characterized by dynamics:

9 $${\tau }_{{\rm{m}}}\frac{{\rm{d}}{x}_{i}}{{\rm{d}}t}=-{x}_{i}\left(t\right)+g\mathop{\sum }\limits_{j=1}^{N}W{p}_{i}\left(t\right)+{w}_{i}s(t)$$

where *τ*_m_ = 20 ms is the membrane time constant, *W* is the synaptic weight matrix, *s* is an input term representing external inputs and *p* represents recurrent inputs. To model spiking, we set a threshold (*θ* = 0.1), such that when the membrane potential *x*_*i*_(*t*) > *θ*, *x*_*i*_(*t*) is set to zero and the instantaneous spiking rate ${r}_{i}\left(t\right)$ is set to 1.

Spiking-evoked input was modelled as a synaptic current with dynamics:

10 $${\tau }_{{\rm{s}}}\frac{{\rm{d}}{p}_{i}}{{\rm{d}}t}=-{p}_{i}\left(t\right)+{r}_{i}\left(t\right),$$

where *τ*_s_ is the synaptic conductance time constant. In excitatory networks, the network time constant *τ*_n_ was derived as $\frac{{\tau }_{{\rm{s}}}}{\left|1-{\lambda }_{\max }\right|}$, where ${\lambda }_{\max }$ is the largest eigenvalue of the synaptic weight matrix *W* (ref. ^40^).

We designed the synaptic connectivity matrix to include a subnetwork of 200 neurons (20% of the network), designated the integration subnetwork as suggested by empirical measurements, with varying densities of random connectivity as highlighted in Fig. 3. Weights of the overall network were sampled from a uniform distribution: ${W}_{{ij}} \sim {U}(\mathrm{0,1}/\sqrt{N})$, while weights of the subnetwork were sampled as ${W}_{{ij}} \sim {U}(\mathrm{0,1}/\sqrt{{N}_{p}})$, where *N*_*p*_ = 200.

External input was provided to the network as a smoothened step function consisting of four pulses at 20 second ISI as provided in vivo. This stimulus drove a random 25% of neurons in the network.

To account for finite-size effects and runaway excitation in networks, we also simulated models with fast feedback inhibition. This was modelled as recurrent inhibition from a single graded input *I*_inh_ representing an inhibitory population that receives equal input from and provides equal input to, all excitatory units. The dynamics of *I*_inh_ evolves as:

11 $${\tau }_{{\rm{I}}}\frac{{\rm{d}}{I}_{{\rm{inh}}}}{{\rm{d}}t}={-I}_{{\rm{inh}}}\left(t\right)+\frac{1}{N}\mathop{\sum }\limits_{n=1}^{N}{r}_{N}(t),$$

where *τ*_I_ = 50 ms is the decay time constant for inhibitory currents. In this model, outside spiking events, the membrane potential evolved as:

12 $${\tau }_{{\rm{m}}}\frac{{\rm{d}}{x}_{i}}{{\rm{d}}t}=-{x}_{i}\left(t\right)+g(\mathop{\sum }\limits_{j=1}^{N}W{p}_{i}\left(t\right)-{g}_{{\rm{inh}}}{I}_{{\rm{inh}}}\left(t\right))+{w}_{i}s(t)$$

Model dynamics were simulated in discrete time using Euler’s method with a timestep of 1 ms and a small Gaussian noise term ${\eta }_{i} \sim N(\mathrm{0,1})/5$ was added at each time step. We used *g* = 1 and varied *g*_inh_ = 1,5,10 as suggested by measurements of inhibitory input to VMHvl^43^.

#### Spatial cluster decoder

To examine whether *x*_1_ and *x*_2_ neurons are spatially clustered in a FOV, we used a linear support vector machine decoder trained to separate cell positions of *x*_1_ and *x*_2_ neurons on each FOV. Shuffled decoder data were generated by randomly assigning neuronal identity. Shuffling was repeated 20 times for each FOV and the performance is reported as the average accuracy of each fit decoder.

#### Decoding behaviour from integration dimension

We trained a frame-wise decoder to discriminate bouts of attack during engaging in aggression from integration dimension activity during observation of attack. We first created ‘trials’ from bouts of attack during observation and engaging in aggression by merging all bouts that were separated by less than 5 s and balancing the data. We then trained a support vector machine to identify a decoding threshold that maximally separates the values of our normalized ‘integration dimension’ signal on frames during observation of aggression versus all other frames and tested the accuracy of the trained decoder on held-out frames. ‘Shuffled’ decoder data were generated by setting the decoding threshold on the same ‘trial’, but with the behaviour annotations randomly assigned to each behaviour bout. We repeated shuffling 20 times. We then tested the decoder trained on data from observation on frames during attack while the animals were engaging in aggression. We report performances of actual and shuffled 1D-threshold ‘decoders’ as the average accuracy score of the fit decoder, on data from all other trials for each mouse. For significance testing, the mean accuracy of the decoder trained on shuffled data was computed across mice, with shuffling repeated 1,000 times for each mouse.

#### Examining the effect of motion on neural encoding during observation of aggression in head-fixed mice

We used an analysis designed to detect motion from video recordings of head-fixed mice^58^. To detect motion this method uses singular value decomposition (SVD) to extract groups of pixels showing high differences in luminance or contrast between consecutive frames. We extracted 500 SVDs from our video recordings that reflect different sources of motion including movements of the limbs, whiskers, nose, ears and more. To predict neural activity from behaviour, we trained generalized linear models to predict the activity of each neuron *k* as a weighted linear combination of the first ten principal components of the 500 SVDs (reflecting over 90% of the SVDs variance) as follows:

$${y}_{k}(t)=\mathop{x}\limits^{\rightharpoonup }(t)\mathop{\beta }\limits^{\rightharpoonup }+\varphi $$

Here, ${y}_{k}(t)$ is the calcium activity of neuron *k* at time *t*, $\mathop{x}\limits^{\rightharpoonup }(t)$ is a feature vector of 10 binary reduced SVD dimensions at time lags ranging from *t* − D to *t* where D = 10s. $\mathop{\beta }\limits^{\rightharpoonup }$ is a behaviour-filter that described how a neuron integrates stimulus over a 10 s period (example filters are shown in Extended Data Fig. 5c). *φ* is an error term. The model was fit using tenfold cross-validation with ridge regularization and model performance is reported as cvR^2^.

### Statistical analysis

Data were processed and analysed using Python, MATLAB and GraphPad (GraphPad PRISM 9). All data were analysed using two-tailed nonparametric tests. Mann–Whitney *U*-tests were used for binary paired samples. Friedman tests were used for non-binary paired samples. Kolmogorov–Smirnov tests were used for non-paired samples. Multiple comparisons were corrected using Dunn’s multiple-comparison correction.

### Reporting summary

Further information on research design is available in the Nature Portfolio Reporting Summary linked to this article.

## Online content

Any methods, additional references, Nature Portfolio reporting summaries, source data, extended data, supplementary information, acknowledgements, peer review information; details of author contributions and competing interests; and statements of data and code availability are available at 10.1038/s41586-024-07915-x.

## Supplementary information

Reporting Summary

## Data Availability

Source data for this Article have been deposited in the DANDI repository with the accession code 001037.

## Code availability

Code for fitting models is available at GitHub (https://github.com/lindermanlab/ssm).
