# Supplementary material for: Causal evidence of a line attractor encoding an affective state
Source: Nature. 2024 Aug 14;634(8035):910–8. doi: 10.1038/s41586-024-07915-x (PMC11499281; doi:10.1038/s41586-024-07915-x)
Supplement: Supplementary file 1 — Reporting Summary [file 41586_2024_7915_MOESM1_ESM.pdf]

nature portfolio

Corresponding author(s): David J Anderson

Last updated by author(s): Jul 29, 2024

Reporting Summary

Nature Portfolio wishes to improve the reproducibility of the work that we publish. This form provides structure for consistency and transparency in reporting. For further information on Nature Portfolio policies, see our Editorial Policies and the Editorial Policy Checklist.

Statistics

For all statistical analyses, confirm that the following items are present in the figure legend, table legend, main text, or Methods section.

n/a	Confirmed	
☐	☒	The exact sample size (*n*) for each experimental group/condition, given as a discrete number and unit of measurement
☐	☒	A statement on whether measurements were taken from distinct samples or whether the same sample was measured repeatedly
☐	☒	The statistical test(s) used AND whether they are one- or two-sided<br>*Only common tests should be described solely by name; describe more complex techniques in the Methods section.*
☐	☒	A description of all covariates tested
☐	☒	A description of any assumptions or corrections, such as tests of normality and adjustment for multiple comparisons
☐	☒	A full description of the statistical parameters including central tendency (e.g. means) or other basic estimates (e.g. regression coefficient) AND variation (e.g. standard deviation) or associated estimates of uncertainty (e.g. confidence intervals)
☐	☒	For null hypothesis testing, the test statistic (e.g. *F*, *t*, *r*) with confidence intervals, effect sizes, degrees of freedom and *P* value noted<br>*Give P values as exact values whenever suitable.*
☒	☐	For Bayesian analysis, information on the choice of priors and Markov chain Monte Carlo settings
☒	☐	For hierarchical and complex designs, identification of the appropriate level for tests and full reporting of outcomes
☒	☐	Estimates of effect sizes (e.g. Cohen's *d*, Pearson's *r*), indicating how they were calculated

Our web collection on statistics for biologists contains articles on many of the points above.

Software and code

Policy information about availability of computer code

Data collection	Calcium imaging data from freely behaving mice was acquired using commercial software from Inscopix, and followed by the MATLAB-based CNMFE package (trace extraction). Calcium imaging data and optogenetic data from two-photon experiments in head-fixed mice was acquired using Bruker's Prairie View software. Streampix software was used to collect behavior data of freely behaving animals and Flycapture was used for behavior recordings of head-fixed animals.
Data analysis	Microendoscopic and two-photos imaging analysis and modeling were performed using custom-written Python (3.8.3) or Matlab (2020a) code. All code used to analyze data in this paper is available in the following GitHub repositories: https://github.com/lindermanlab/ssm. Statistics was computed using the Prism software and Adobe Illustrator was used for assembling figures.

For manuscripts utilizing custom algorithms or software that are central to the research but not yet described in published literature, software must be made available to editors and reviewers. We strongly encourage code deposition in a community repository (e.g. GitHub). See the Nature Portfolio guidelines for submitting code & software for further information.

Data

Policy information about availability of data

All manuscripts must include a data availability statement. This statement should provide the following information, where applicable:

- Accession codes, unique identifiers, or web links for publicly available datasets
- A description of any restrictions on data availability
- For clinical datasets or third party data, please ensure that the statement adheres to our policy

Behavioral and imaging data have been made available in the DANDI repository, accession number: 001037

Research involving human participants, their data, or biological material

Policy information about studies with human participants or human data. See also policy information about sex, gender (identity/presentation), and sexual orientation and race, ethnicity and racism.

Reporting on sex and gender	N.A
Reporting on race, ethnicity, or other socially relevant groupings	N.A
Population characteristics	N.A
Recruitment	N.A
Ethics oversight	N.A

Note that full information on the approval of the study protocol must also be provided in the manuscript.

Field-specific reporting

Please select the one below that is the best fit for your research. If you are not sure, read the appropriate sections before making your selection.

☒ Life sciences ☐ Behavioural & social sciences ☐ Ecological, evolutionary & environmental sciences

For a reference copy of the document with all sections, see nature.com/documents/nr-reporting-summary-flat.pdf

Life sciences study design

All studies must disclose on these points even when the disclosure is negative.

Sample size	No sample-size calculations were performed; sample sizes were selected based on prior experience with similar behavior assays and imaging datasets (Remedios and Kennedy et al., 2017, Karigo et al., 2020 and Yang et al., 2022).
Data exclusions	Mice with mislocation of viral injections and GRIN lens implantation were excluded from analysis.
Replication	All experiments were repeated at least twice with separate cohorts of animals and reproducibility was confirmed.
Randomization	For imaging experiments, animals were randomly chosen from the Esr1 transgenic cohort.
Blinding	No blinding for performed in this study.

Reporting for specific materials, systems and methods

We require information from authors about some types of materials, experimental systems and methods used in many studies. Here, indicate whether each material, system or method listed is relevant to your study. If you are not sure if a list item applies to your research, read the appropriate section before selecting a response.

Materials & experimental systems

n/a	Involved in the study
☒	☐ Antibodies
☒	☐ Eukaryotic cell lines
☒	☐ Palaeontology and archaeology
☐	☒ Animals and other organisms
☒	☐ Clinical data
☒	☐ Dual use research of concern
☒	☐ Plants

Methods

n/a	Involved in the study
☒	☐ ChIP-seq
☒	☐ Flow cytometry
☒	☐ MRI-based neuroimaging

Animals and other research organisms

Policy information about studies involving animals; ARRIVE guidelines recommended for reporting animal research, and Sex and Gender in Research

Laboratory animals	Study used male Esr1 mice aged 2 months for neural recording experiments. Male BALB/c and Swiss Webster mice were used for aggression assays. All mice in this study, including wild-type and transgenic mice, were bred at Caltech. All mice were housed in ventilated micro-isolator cages in a temperature-controlled environment (median temperature 23°C, humidity 60%), under a reversed 11-h dark–13-h light cycle, with ad libitum access to food and water. Mouse cages were changed weekly.
Wild animals	N.A
Reporting on sex	Only male mice are used for neural recording experiments in the study.
Field-collected samples	N.A
Ethics oversight	All experimental procedures involving the use of live animals or their tissues were performed in accordance with the NIH guidelines and approved by the Institutional Animal Care and Use Committee (IACUC) and the Institutional Biosafety Committee at the California Institute of Technology (Caltech).

Note that full information on the approval of the study protocol must also be provided in the manuscript.

Plants

Seed stocks	N.A
Novel plant genotypes	N.A
Authentication	N.A
